# Advanced interpretable diagnosis of Alzheimer's disease using SECNN-RF framework with explainable AI

**DOI:** 10.3389/frai.2024.1456069

**Published:** 2024-09-02

**Authors:** Nabil M. AbdelAziz, Wael Said, Mohamed M. AbdelHafeez, Asmaa H. Ali

**Affiliations:** ^1^Information System Department, Faculty of Computers and Informatics, Zagazig University, Zagazig, Egypt; ^2^Computer Science Department, Faculty of Computers and Informatics, Zagazig University, Zagazig, Egypt

**Keywords:** Explainable Artificial Intelligence (XAI), Squeeze-and-Excitation (SE block), Squeeze-and-Excitation Convolutional Neural Network with Random Forest (SECNN-RF), explainability analysis, saliency map

## Abstract

Early detection of Alzheimer's disease (AD) is vital for effective treatment, as interventions are most successful in the disease's early stages. Combining Magnetic Resonance Imaging (MRI) with artificial intelligence (AI) offers significant potential for enhancing AD diagnosis. However, traditional AI models often lack transparency in their decision-making processes. Explainable Artificial Intelligence (XAI) is an evolving field that aims to make AI decisions understandable to humans, providing transparency and insight into AI systems. This research introduces the Squeeze-and-Excitation Convolutional Neural Network with Random Forest (SECNN-RF) framework for early AD detection using MRI scans. The SECNN-RF integrates Squeeze-and-Excitation (SE) blocks into a Convolutional Neural Network (CNN) to focus on crucial features and uses Dropout layers to prevent overfitting. It then employs a Random Forest classifier to accurately categorize the extracted features. The SECNN-RF demonstrates high accuracy (99.89%) and offers an explainable analysis, enhancing the model's interpretability. Further exploration of the SECNN framework involved substituting the Random Forest classifier with other machine learning algorithms like Decision Tree, XGBoost, Support Vector Machine, and Gradient Boosting. While all these classifiers improved model performance, Random Forest achieved the highest accuracy, followed closely by XGBoost, Gradient Boosting, Support Vector Machine, and Decision Tree which achieved lower accuracy.

## 1 Introduction

Early and accurate diagnosis of Alzheimer's Disease (AD), the most prevalent form of dementia demanding significant medical support, is crucial for initiating clinical progress and ensuring effective patient treatment (Liu et al., 2014). This chronic neurodegenerative disorder progressively damages brain cells, leading to memory and thinking impairments, ultimately impeding even basic activities (Przedborski et al., 2003). In its early stages, neuroimaging and computer-aided tools aid clinicians in accurately classifying AD (Giorgio et al., 2020).

Artificial Intelligence (AI) is a subdivision of computer science that has transformed how individuals carry out their daily activities through the use of machines that require minimal human involvement, thereby enabling automated and intelligent actions. AI is considered an incredible prospect for resolving neurology disease issues, generating additional perspectives, and enhancing the quality of decision support. AI and Machine Learning (ML) are already revolutionizing several medical systems, with further advancements expected in the future (Bai et al., 2021). The majority of AI algorithms have been referred to as “black boxes” by researchers due to their intricate and virtual nature, making them challenging to explain and justify to individuals. A black box concept is one where the inputs and outputs are known, but you are unable to determine how the outputs are produced from the inputs. Developers are also unable to explain why the model has reached a particular conclusion or which factors were taken into consideration when making a decision. This is due to the models' intricate internal structure, and the poor offer of interpretability. The consideration of complicated models' ambiguous nature has limited their potential use in making key decisions, such as those involving medical procedures that could endanger people's lives and health. Users can accept or reject forecasts and recommendations based on the justification behind the predictions made by interpretable ML systems (Ahmad et al., 2018). The existence of this particular obscurity has led to a demand for algorithms in the field of Explainable Artificial Intelligence (XAI) (Wachter et al., 2017).

XAI has been designed to explain its purpose, perception, and process decision in terms that the common person can understand. The concept behind XAI is that AI algorithms and systems shouldn't be “mysterious models” that are incomprehensible to humanity (Pawar et al., 2020; Giuste et al., 2022). The patient's AD prognoses are based on his current lifestyle and family medical history indicators, which are time-varying, multi-connected, and non-linear. Early AD detection is frequently essential to patients' recovery or to stop the disease from progressing to more severe stages. The situation is critical since it impacts patient health and relies on current prediction outputs for better decisions. Transparency and trust among doctors and other medical professionals are necessary for neurology diseases AI applications to be accepted and integrated into practical implementation. Motivated by the need for accurate and interpretable diagnosis in neurodegenerative diseases like Alzheimer's, this paper presents a novel hybrid framework that leverages the strengths of both deep learning and traditional machine learning.

Our approach methodically analyzes AD image data (specifically MRI images of size 128 × 128 × 3). This study achieves exceptional classification accuracy by combining the feature extraction capabilities of a Squeeze-and-Excitation Convolutional Neural Network (SECNN) with the decision-making power of a Random Forest classifier (SECNN-RF). Furthermore, this study incorporates the SECNN model to refine extracted features and facilitate explainability through insightful attention weights. This unique collaboration not only surpasses state-of-the-art performance in accuracy but also provides valuable insights into the reasoning behind model predictions. This comprehensive and interpretable approach represents a significant step toward enhancing diagnosis and potential treatment development for AD.

This paper presents a novel SECNN-RF framework that significantly enhances Alzheimer's Disease (AD) detection from MRI scans by integrating adaptive attention mechanisms and leveraging ensemble learning techniques. Key innovations include:

- The framework integrates SE blocks within each convolutional layer of the CNN to apply an adaptive attention mechanism. This recalibrates channel-wise feature responses, allowing the network to dynamically prioritize the most informative features for Alzheimer's Disease (AD) detection from MRI scans. This framework reduces trainable parameters, making the model suitable for medical applications with limited computational resources.
- Unlike traditional CNN architectures that use a SoftMax layer for classification, the SECNN-RF framework employs a Random Forest classifier. This replacement leverages the robust, ensemble learning capabilities of Random Forests, improving the model's classification accuracy for AD diagnosis. The isolation between the feature extraction and classification decisions makes our model more computationally efficient.
- To promote trust in the decisions made by our model, the proposed method integrates saliency maps to generate visual explanations of classification decisions made by the model enhancing the transparency of the model and making it easy for clinicians to interpret and verify the results.

The remainder of this work is arranged subsequently. Section 2 presents a literature review. Section 3 delves into the proposed model. The experimental results are discussed in Section 4. Section 5 presents a discussion and explanation of the results. Finally, this study concludes in Section 6.

## 2 Literature review

This section provides a summary of the various techniques utilized for AD using medical imaging. This paper categorizes these methods into two groups: the first category delves into deep learning approaches, while the second category focuses on Interpretable deep learning for AD. XAI is a new field of research in ML that examines how AI systems react to black-box decisions (Saraswat et al., 2022). Transparency in ML and DL algorithms involves explaining their outcomes and decisions, which can be achieved by developing interpretable models, methods, and interfaces to provide human-understandable explanations for their behavior (Abujabal et al., 2017). Interpretable ML systems provide users with explanations for accepting or rejecting predictions and recommendations, thereby enabling them to understand the reasoning behind these outcomes (Adadi and Berrada, 2018).

### 2.1 Deep learning methods

Different deep-learning techniques have been used in multiple studies to categorize neurological diseases. For the following reasons, convolutional neural networks (CNNs) are widely employed in image-based neurological disease diagnosis: (1) They can process a great deal of contextual data, and abnormal information. (2) The input image connections are used hierarchically, with the processing taking the use of spatial relationships throughout. (3) Because they use unique pooling, parameter sharing, and convolution processes, they are also computationally efficient. CNNs succeed at producing better outcomes than pre-trained models, making them ideal for generating customized models.

AbdulAzeem et al. (2021) created a modified CNN with five layers for AD categorization. This work processed the images using data augmentation and adaptive thresholding. For AD classification, Katabathula et al. introduced a dense CNN architecture that combines hippocampal segmentation and global shape representations (Katabathula et al., 2021). Raju et al. (2020) classified AD using an SVM with an RBF kernel after extracting visual characteristics using a particular 3D CNN architecture. Single-modality data can only identify a handful of the degenerative changes associated with AD, thereby limiting the classifier's accuracy. Extensive research has been conducted to develop multimodal classification algorithms. Huang et al. (2019) developed a VGG-like network for multimodal AD classification. For imaging data, 3D-CNNs are used, and stacks of denoise auto-encoders are used for clinical and genetic data. Based on the Alzheimer's Disease Neuroimaging Initiative (ADNI) dataset, the authors show that deep models outperform shallow models such as decision trees, Support Vector Machines (SVM), K-neighbors, and random forests (Venugopalan et al., 2021). The paper presents the results and analyses of the detection of dementia using a variety of ML models as well as the use of shallow learning models to identify and forecast AD. The SVM was the approach that performed the best in this case, with an accuracy of roughly 92.0% (Bari Antor et al., 2021). 3D brain Magnetic Resonance Imaging (MRI) can be used to diagnose AD. By utilizing the Gaussian Mixture Model (GMM) as an additional input to CNN, XGBoost, and SVM for AD classification. The dataset accuracy reached 88% and 80% (Tuan et al., 2020). Savaş (2022) introduced a study that involved evaluating various pre-trained networks, including EfficientNetB0, ResNet-50, DenseNet, Xception, etc., for Alzheimer's disease (AD) classification. The findings indicated that EfficientNetB0 exhibited a slightly superior performance compared to the other architectures.

Muruganm et al. (2021), proposed DEMNET, a deep-learning model for diagnosing AD from MRI images. They employed preprocessing, oversampling, and data splitting before feeding the data to DEMNET for feature extraction and classification. This yielded a 95.23% accuracy in multi-class classification. Loddo et al. (2022), proposed a fully automated ensemble model using pre-trained deep models (AlexNet, ResNet-101, InceptionResNetV2) for AD diagnosis from MRI images. Notably, they achieved high accuracies (96.57% binary, 97.7% multi-class) without image preprocessing. Employing a first-stage preprocessing followed by parallel processing through pre-trained DenseNet201 and DenseNet121 models, Sharma et al. (2022), developed “HTLML” for multi-class AD detection from MRI images. Each pre-trained model utilized separate classifiers, with their outputs combined via voting for a final decision. While achieving an impressive 91.75% accuracy, the study did not leverage data augmentation and relied on relatively small datasets, limiting potential generalizability. Mohammed et al. (2021), proposed a hybrid model featuring image preprocessing, a CNN for feature extraction, and an SVM for final classification, achieving 94.80% accuracy in multi-class AD diagnosis from MRI. Balasundaram et al. (2023), achieved 94.1% accuracy in multi-class AD diagnosis using a pre-trained ResNet50 model on MRI images, but relied solely on basic preprocessing and a single model, potentially limiting further performance gains.

Bangyal et al. (2022), demonstrated the superiority of deep learning for AD detection in MRI images compared to traditional methods, achieving 94.63% multi-class accuracy. To accurately classify early Alzheimer's stages and reduce computational costs, a novel model named DAD-Net was developed by Ahmed et al. (2022). Addressing the class imbalance in the Kaggle dataset through synthetic oversampling, DAD-Net achieves outstanding performance score, precision, and recall compared to DEMENET and CNN models. Ahmed et al. proposed a lightweight deep learning architecture that combines feature extraction and classification into a single stage, eliminating the need for deeper layers and traditional methods. This efficient approach resulted in a seven-layer model (imagine a compact, yet powerful AI) that achieved an impressive accuracy of 99.22% for binary classification and 95.93% for multi-classification (El-Latif et al., 2023). Abbas et al. (2023), developed a new CAD-ALZ approach using ConvMixer layers with a blockwise fine-tuning strategy on a small dataset. Data augmentation increases the dataset size, and robust features are detected through the ConvMixer model, followed by classification with a random forest. The CAD-ALZ model shows excellent performance across six evaluation metrics, achieving 99.69% sensitivity and a 99.61% F1-score. In AlSaeed and Omar (2022), the authors proposed ResNet50, a pre-trained CNN deep learning model, as an automatic feature extraction method for identifying Alzheimer's disease from MRI scans. The performance of a CNN using standard Softmax, SVM, and RF was then tested using several metric metrics, including accuracy. The results demonstrated that our model beat other cutting-edge models by achieving better accuracy, with an accuracy range of 85.7, 92, and 99% for softmax, SVM, and random forest models, respectively, using the MRI ADNI dataset.

### 2.2 Interpretable deep learning methods

The most recent XAI systems that are related to the neurology diseases field are presented in this section. According to the authors in Zhang et al. (2022), various XAI-enabled methods for medical diseases and XAI applications were described, along with recent and present trends in medical diagnosis and application using XAI based on findings from various research platforms. Finally, the research directions and challenges achieved were discussed. In Amann et al. (2020), authors discussed XAI in healthcare in a multidisciplinary way to examine its importance from a legal, patient, medical, and technological perspective. The importance of XAI in the clinical system from an ethical and personal perspective is concluded by the authors after deducing a set of results for the applicability of views. In Yang et al. (2022), the authors introduced an overview of current XAI developments and recent advancements in healthcare applications. Through the use of two descriptive clinical-level case studies, the authors demonstrate how XAI makes use of multi-modal and multi-center data fusion. According to Tjoa and Guan (2020), ML algorithms are interpretable and explainable, but the authors identify open challenges and opportunities in the medical context by analyzing their interpretability and explainability into two categories: perceptive interpretation and mathematical structural interpretation. The study in Moradi and Samwald (2022) discusses already-developed AI methods, such as ML/DL, and expands the survey to discuss the implications of XAI in biomedical and future medical applications. In Böhle et al. (2019), authors proved that to better explain AD classification by applying Layer-wise Relevance Propagation (LRP) for showing CNN decisions, researchers turned to the ADNI MRI dataset. LRP was proven to apply to the diagnosis of comparable diseases using MRI data, and it was shown to be useful for the explainability of classification predictions in AD. In Kim et al. (2021), Graph Neural Networks (GNNs) were used to classify AD and Mild Cognitive Impairment (MCI). GNNExplainer demonstrates nodes of relevance with a high region of interest, signifying a significant improvement in categorization. The authors discovered that GNNExplainer produces interpretable outputs. Furthermore, the explainer can record the predictor's neuro-anatomical contribution, providing additional biological explanations to well-understand AD change. The authors believe that the GNNExplainer is useful because it surpasses other competing models in terms of prediction accuracy (e.g., DNN, SVM). A cognitive signature based on DL was developed for Parkinson's and Alzheimer's disease brain Positron Emission Tomography (PET) scans. The CNN model generated 128 features for each sample, which were compared using t-stochastic Neighbor Embedding (t-SNE). Using imaging biomarkers enables an objective valuation of cognitive decline, as demonstrated (Choi et al., 2020). Multiple sclerosis was classified using 3D CNN on an MRI dataset, and LRP was utilized to validate the model's decisions. The results of the research indicated that the utilization of the framework and LRP resulted in enhanced comprehensibility of the model's decision-making process (Eitel et al., 2019).

In conclusion, existing techniques encounter key limitations including:

- Many deep learning models, particularly those utilizing 3D-CNNs, require significant computational resources for training and inference. This includes high memory usage and processing power, which may not be feasible for deployment in resource-constrained medical settings or smaller clinics.
- Due to the high complexity and number of parameters in deep learning models, there is a risk of overfitting, particularly with small datasets.
- While some studies have incorporated explainable AI (XAI) techniques, many deep learning models still function as “black boxes.” This lack of interpretability can be a significant drawback in clinical settings where understanding the model's decision-making process is crucial.

Toward this end, this study proposes a novel SECNN-RF framework to address key limitations in the existing literature on Alzheimer's Disease (AD) diagnosis using medical imaging. The SECNN-RF framework is designed to minimize trainable parameters while maintaining high performance, making it efficient for medical imaging applications. The model employs a CNN with multiple convolutional layers enhanced with SE attention blocks. This mechanism allows the model to focus on the most informative features, enhancing its ability to distinguish between subtle differences in medical images. In addition, Regularization is achieved through Dropout layers after each SE-enhanced convolutional layer to prevent overfitting and ensure that the model maintains high accuracy without being overly sensitive to the training data. In the final stage, high-dimensional feature vectors are extracted and flattened, then classified using a Random Forest (RF) classifier. The RF classifier aggregates predictions from multiple decision trees, enhancing interpretability and robustness. This combination ensures that the SECNN-RF framework not only achieves high accuracy but also provides transparency and reliability, crucial for clinical decision-making. By leveraging the SECNN to extract important features from MRI scans and employing a Random Forest classifier for the classification step, the proposed model offers an efficient, interpretable, and robust solution. This study demonstrates that SECNN-RF not only achieves high accuracy but also addresses issues such as computational efficiency, class imbalance, and the need for interpretability in clinical decision-making

## 3 Proposed model

**Figure 1:**
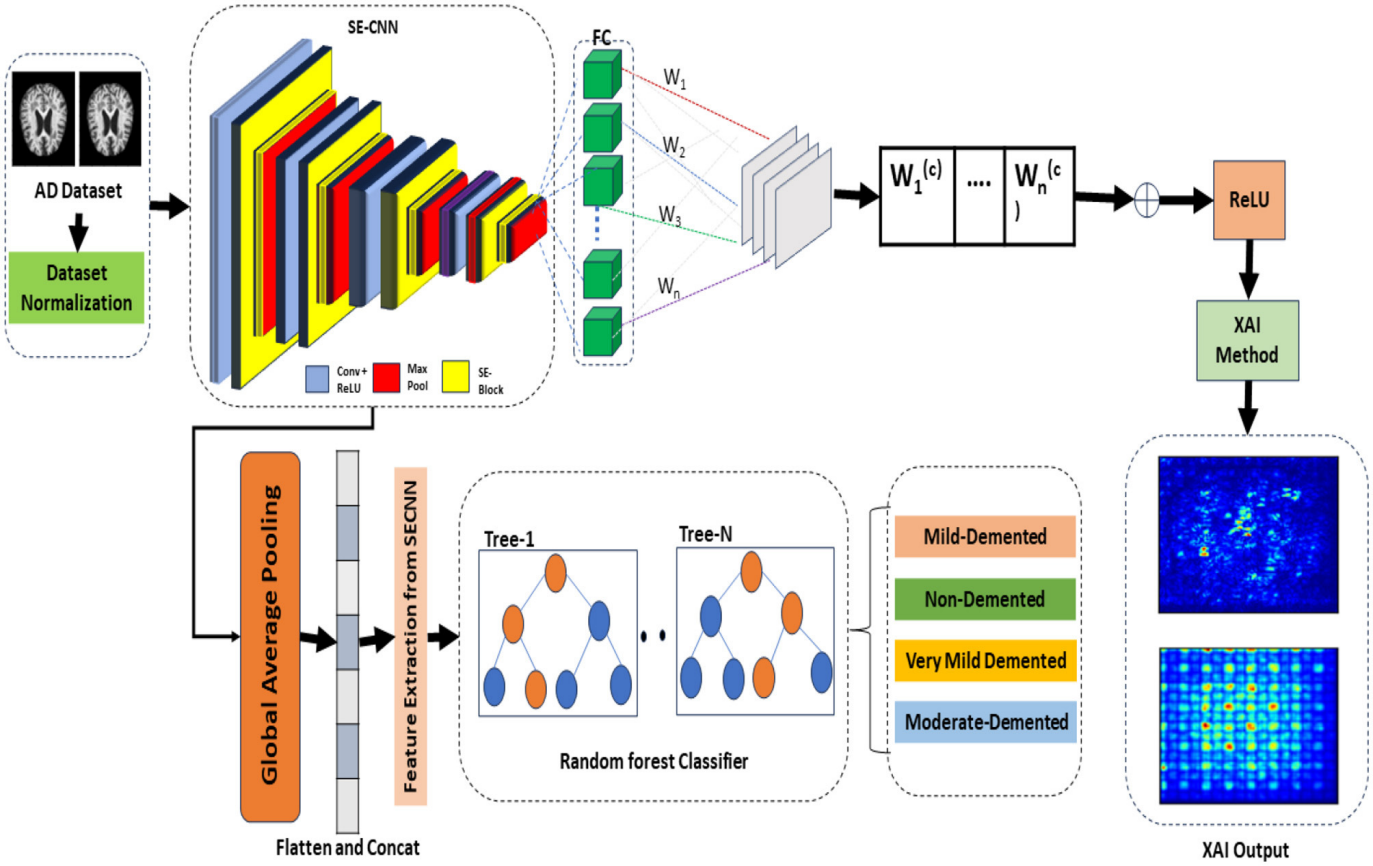
The proposed model visualization for early detection and explanation of AD.

The proposed framework termed the Squeeze-and-Excitation Convolutional Neural Network with Random Forest (SECNN-RF), is shown in Figure 1. SECNN-RF is designed to minimize trainable parameters while maintaining high performance, making it efficient for medical imaging applications. The model undergoes preprocessing steps such as resizing and normalization to standardize MRI inputs. During classification, the CNN extracts deep features enhanced by SE blocks and regularized by Dropout. These features are then classified by the Random Forest, providing transparent and explainable results, which are crucial for medical applications where understanding the rationale behind model predictions aids clinicians in decision-making. A distinctive aspect of the proposed framework is its use of the Squeeze-and-Excitation Convolutional Neural Network (SECNN) to extract important features from MRI scans. Instead of a traditional SoftMax final layer, the model employs a Random Forest classifier for the classification step. The detailed architecture of the proposed model is presented in Supplementary Table 1. This table includes the layer type, the output data shapes after each layer, the number of parameters for each layer, and the connections between layers. Algorithm 1 formalizes the SECNN-RF proposed model.

**Algorithm 1:**
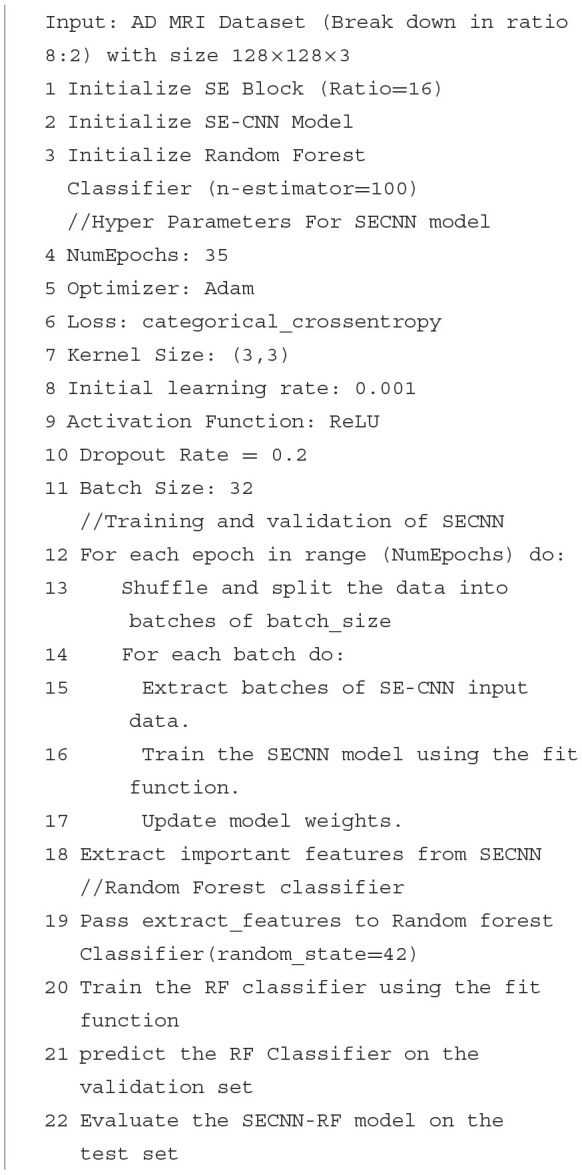
The proposed model (SECNN-RF).

### 3.1 Preprocessing and dataset preparation

#### 3.1.1 Image preprocessing

The input MRI images are standardized through resizing and normalization to ensure consistency and stability in the model's performance.

- **Resizing:** Each input image is resized to 128 × 128 × 3. Although grayscale images with the dimension (128 × 128 × 1) reduce the computation complexity, in most cases, the CNN model achieves higher performance in multi-class classification using RGB images with the dimension (128 × 128 × 3) compared to grayscale images (Kim et al., 2020). Our goal was to achieve good performance with low computational complexity, so we used RGB images to achieve high performance.
- **Rescaling**: The input was changed from [0, 255] to [0, 1] by setting the scale to 1/255.
- **Augmentation:** Various image augmentation techniques are applied, including zooming within the range of 0.99–1.01, brightness adjustment between 0.8 and 1.2, and horizontal flipping.
- **Class imbalance:** To address the issue of class imbalance, we applied the Synthetic Minority Oversampling Technique (SMOTE) method to generate synthetic samples, resulting in a balanced training dataset is 8,192 images including 2,048 images for each class.

### 3.2 Feature extraction and attention mechanism

#### 3.2.1 Convolutional neural network

The CNN forms the backbone of our feature extraction process, designed to automatically and adaptively learn spatial hierarchies of features from the input data.

• Convolutional layers: each convolutional layer applies a set of filters to the input image or feature map to extract various features. Mathematically, the convolution operation at layer L for an input x with a filter f is given by:
(1) $$\begin{array}{l} Y_{\left(i,j,c,k\right)}=\overset{C}{\underset{c=1}{\sum }}\overset{M}{\underset{m=1}{\sum }}\overset{N}{\underset{n=1}{\sum }}X_{\left(i+m-1,j+n-1,c\right)}\times F_{\left(m,n,c,k\right)} \end{array}$$

where *Y*_(*i, j, c, k*)_ denote output feature map in spot ( *i, j*) in the *k*−*th* output channel. *X*_(*i*+*m*−1, *j*+*n*−1, *c*)_ denote input feature map in spot (i+m-1, *j*+*n*−1 ) in the c-th input channel. *F*_(*m, n, c, k*)_ represent filter (kernel) value at position (*m, n*) for the c-th input channel and the *k*-th output channel. Symbols *M* and *N* represent the height and width of the convolution filter (kernel), with *C* representing the total number of input channels. The symbols *i, j* denote starting indices for the current position in the input feature map, while *k* denotes the output channel index.

#### 3.2.2 Adaptive attention with Squeeze-and-Excitation blocks

**Figure 2:**
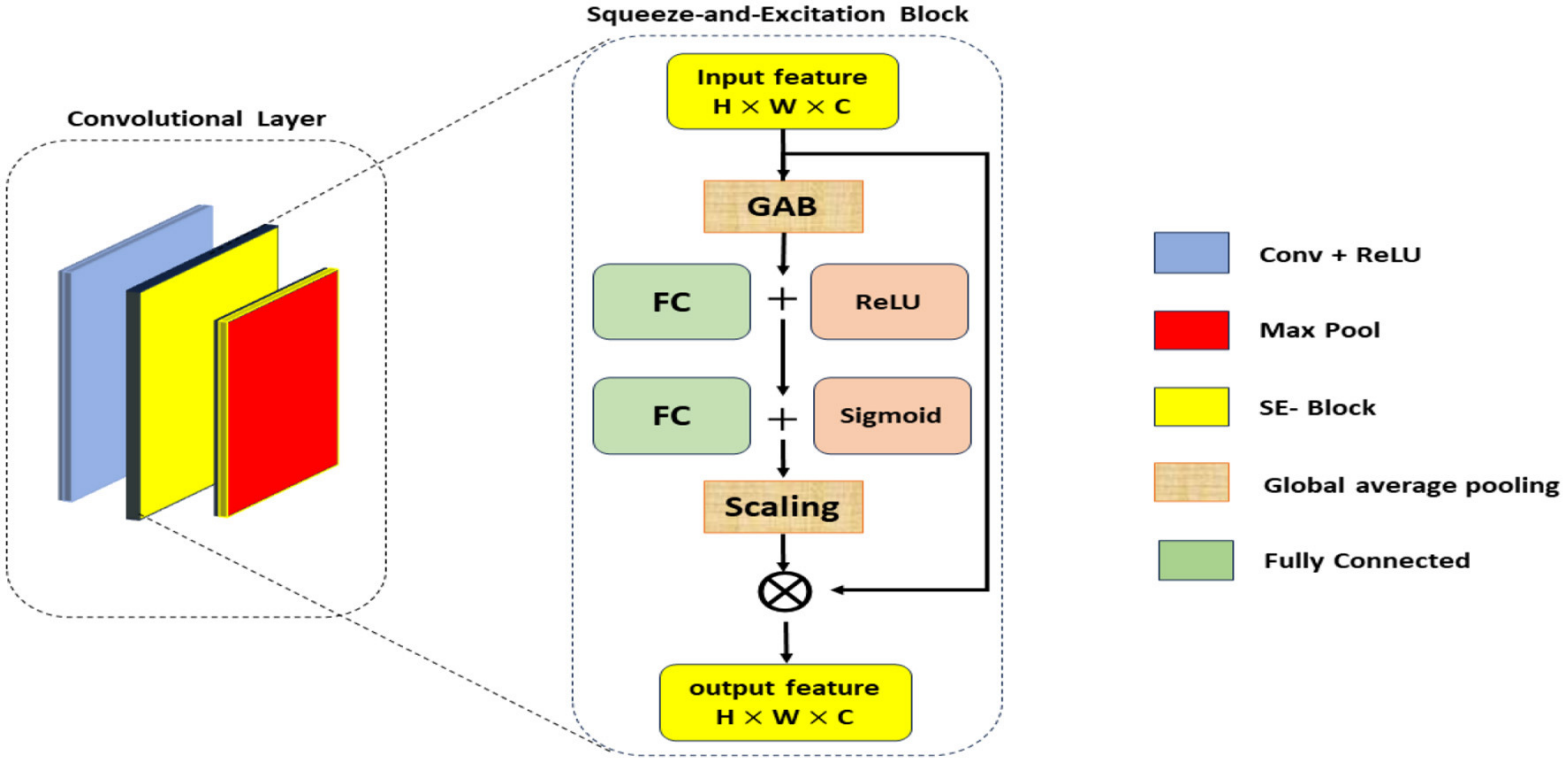
The architecture of Squeeze and Excitation SE-block.

The SE blocks enhance the CNN's ability to focus on critical features by reweighting the channel-wise feature responses. The SE blocks introduce an adaptive mechanism that recalibrates channel-wise feature responses, enhancing the network's ability to emphasize the most informative features while suppressing less relevant ones. Unlike traditional pooling and activation layers, which treat channels independently and lack this adaptive capability, SE blocks dynamically adjust feature weights based on their importance, leading to improved representational power and model performance. This adaptive recalibration helps capture subtle patterns in the data, making SE blocks particularly effective for tasks such as Alzheimer's Disease detection from MRI scans, where accurate and detailed feature representation is crucial. As illustrated in Figure 2, the SE block comprises a global average pooling layer followed by two fully connected layers. The first fully connected layer employs a ReLU activation function, while the second layer uses a sigmoid activation function, enabling the block to effectively recalibrate channel-wise feature responses (Hu et al., 2018).

• Squeeze operation: global average pooling is applied to the input feature map X to generate channel-wise statistics:
(2) $$\begin{array}{l} Z_{c}=\frac{1}{H\;\times W}\overset{H}{\underset{i=1}{\sum }}\overset{W}{\underset{j=1}{\sum }}X_{i,j,c} \end{array}$$
  where Z_c_ is the c-th element of the squeezed feature vector, and *H* and *W* are the spatial dimensions of the input.
• Excitation operation: The squeezed vector is passed through two fully connected (FC) layers with ReLU and sigmoid activations to produce the reweighting coefficients **s**:
(3) $$\begin{array}{l} s=\;\sigma \left(W_{2}\cdot ReLU\left(W_{1}\cdot Z\right)\;\right) \end{array}$$
  where *W*_1_ and *W*_2_ *a*re the weights of the FC layers, and *σ* denotes the sigmoid function.
• Reweighting: The original feature map X is scaled by the coefficients s to produce the recalibrated feature map $\hat{X}$**:**
(4) $$\begin{array}{l} {\hat{X}}_{i,j,c}=X_{i,j,c}\times \;s_{c} \end{array}$$

#### 3.2.3 Regularization with dropout layers

To prevent overfitting and improve the model's generalization, Dropout layers are included after each SE-enhanced convolutional layer.

• Dropout operation: during training, each neuron is randomly set to zero with a probability *p*. This process can be mathematically represented as:
(5) $$\begin{array}{l} y=x\;\cdot mask\;where\;mask\;\sim \;Bernoulli\left(p\right) \end{array}$$

where y is the output after dropout, x is the input, and mask is a binary mask sampled from a Bernoulli distribution.

### 3.3 Feature extraction from SECNN

The processes of extracting important features from the SECNN model are discussed in the following steps:

#### 3.3.1 Convolutional feature extraction

- SECNN model: the SECNN model comprises several convolutional layers, SE blocks, and pooling layers that learn and recalibrate features from the input MRI images. These layers are designed to capture hierarchical patterns and important spatial features relevant to Alzheimer's diagnosis.
- Intermediate feature maps: during the forward pass of the SECNN, feature maps are generated at various intermediate layers. These feature maps contain valuable information about the input data at different levels of abstraction.

#### 3.3.2 Global average pooling

- Pooling layer: to create a fixed-size feature vector from the variable-size intermediate feature maps, we apply a Global Average Pooling (GAP) layer. This layer averages each feature map across its spatial dimensions, producing a single value for each feature map.
- Resulting feature vector: the output of the GAP layer is a 1-dimensional feature vector that represents the most salient features learned by the SECNN model. This vector encapsulates the high-level information necessary for classification tasks.

#### 3.3.3 Feature extraction for random forest classifier

- Extraction process: the 1-dimensional feature vector obtained from the GAP layer is extracted from the SECNN model and used as input for the RF classifier.

### 3.4 Replacing SoftMax with random forest classifier

Instead of using a dense layer with a SoftMax activation for classification, the SECNN-RF framework employs a Random Forest (RF) classifier for the final decision-making step.

• Feature extraction: the SECNN, up to the GlobalAveragePooling2D and Flatten layers, is used to extract a high-dimensional feature vector from the input image.
• Random forest classifier: the feature vectors serve as input to the RF classifier. The RF model, composed of an ensemble of decision trees, aggregates the predictions from each tree to make a robust final classification.
(6) $$\begin{array}{l} RF\left(x\right)=\;\frac{1}{N}\;\overset{N}{\underset{i=1}{\sum }}T_{i}\left(x\right) \end{array}$$

where *T*_*i*_ denotes the i-th decision tree, and N is the total number of trees in the forest that is equal to 100 trees in our proposed model.

### 3.5 Explainable model design

The saliency map technique involves calculating gradients of the loss function concerning all network weights and backpropagating these gradients to the input data layer. This process highlights the regions of the input image that contribute the most to the assigned class, providing valuable insights into the model's decision-making process. If input features are denoted as *x* and the score for predicting class c as Sc, then the map of the contribution score is constructed as follows:


(7) $$\begin{array}{l} L_{Saliency\;map}^{c}=\;\frac{\partial S^{c}}{\partial x} \end{array}$$

## 4 Results and discussions

### 4.1 Dataset description

Several datasets are available online for Alzheimer's Disease (AD) classification, including those from ADNI and OASIS, which are 5 and 1.5 GB, respectively. In contrast, the Kaggle dataset we used to be only 36 MB, making it much more manageable. This simplicity facilitated a more efficient workflow and quicker experimentation process. The Kaggle dataset is easily accessible, free, and well-organized with different classes. Its reasonable size and preprocessed nature make it ideal for our research. Based on these factors, we chose to use the Kaggle dataset. The Kaggle dataset already comprises pre-selected 2D slices, which simplifies the preprocessing pipeline and focuses the analysis on clinically relevant sections of the brain. Also, using 2D slices reduces the computational complexity compared to 3D volumetric data, enabling faster training and inference while still providing sufficient information for Alzheimer's diagnosis. To rigorously assess the efficacy of our proposed model, we curated a comprehensive dataset of AD-MRI images. This collection was meticulously sourced from the Kaggle platform, specifically the “Alzheimer's Dataset: 4 Class of Images” (https://www.kaggle.com/datasets/tourist55/alzheimers-dataset-4-class-of-images). The dataset encompasses a diverse spectrum of AD severity, encompassing four distinct classes: very mild demented, moderate demented, mild demented, and no demented. This comprehensive representation of disease progression is paramount for robust model development. The dataset has an image size of 176 × 208. The images are resized into 128 × 128. From the original 6,400 MRI images, we judiciously selected 5,121 images for training purposes, while the remaining images were reserved for rigorous testing. The precise distribution of images across each class within the training and testing sets is meticulously detailed in Table 1, ensuring transparency and reproducibility in our experimental setup.

**Table 1:** MRI image description for each class of AD dataset.

	**Very mild demented**	**Moderate demented**	**Mild demented**	**Non-demented**
Training	1,792	52	717	2,560
Testing	448	12	179	640

### 4.2 Preprocessing step

The AD dataset was rescaled from (0, 255) to (0, 1) by setting the scale to 1/255. Various image augmentation techniques were applied to the training dataset, including zooming within the range of 0.99–1.01, brightness adjustment between 0.8 and 1.2, and horizontal flipping. To address the class imbalance, the SMOTE method was used to generate synthetic samples, resulting in a balanced training dataset of 8,192 images, with 2,048 images for each class. The data was systematically divided into training (0.8) and validation (0.2) sets to evaluate the model's performance effectively as shown in Table 2. The data is resized to the following dimensions 128 × 128.

**Table 2:** Description of AD dataset before and after SMOTE method.

	**Before SMOTE**	**After SMOTE**			**Testing**
	**Training**	**Training**			
		**Training**	**Validation**	**Total**	
Mild demented	717	1,638	410	2,048	179
Moderate demented	52	1,637	411	2,048	12
Non-demented	2,560	1,639	409	2,048	640
Very mild demented	1,792	1,639	409	2,048	448
Total	5,121	6,553	1,639	8,192	1,279

### 4.3 Implementation setup

This study runs experiments using TensorFlow 2.15.0 in a Python 3.11 virtual environment for building DL models. All experiments are conducted on a Dell workstation with 16 GB RAM and Intel ^®^ Xe^®^(R) CPU E5-2670 @ 2.60 GHz. NVIDIA Quadro P2000 8GB GPUs accelerated models training.

### 4.4 Evaluation metrics

Popular multi-class classification metrics as given in Equations 8–11 developed as a function of true positive (TP), false positive (FP), false negative (FN), and true negative (TN) were used to evaluate the detection performance of competing methods.


(8) $$\begin{array}{l} Accuracy\;\left(ACC\right)\;=\;\frac{TP+TN}{TP+FP+TN+FN} \end{array}$$

(9) $$\begin{array}{l} Precision\;\left(P\right)\;=\;\frac{TP}{TP+FP} \end{array}$$

(10) $$\begin{array}{l} Recall\;=\frac{TP}{TP+FN} \end{array}$$

(11) $$\begin{array}{l} F1-Score\;=\frac{2TP}{2TP+FP+FN} \end{array}$$

### 4.5 Experiments

The following experiments were conducted with an initial learning rate of 0.001 over 35 epochs, using the hyperparameters detailed in Table 3. The Kaggle dataset was split into training and testing sets with proportions of 80 and 20%, respectively.

**Table 3:** Hyperparameters for experiment models.

**Hyper parameters**	**Description**
Num of epochs	35
Initial learning rate	0.001
Kernel size	(3.3)
Optimizer	Adam
Loss	Categorical_crossentropy
Activation function	ReLU
Batch size	32

#### 4.5.1 Experiment 1: CNN model

**Figure 3:**
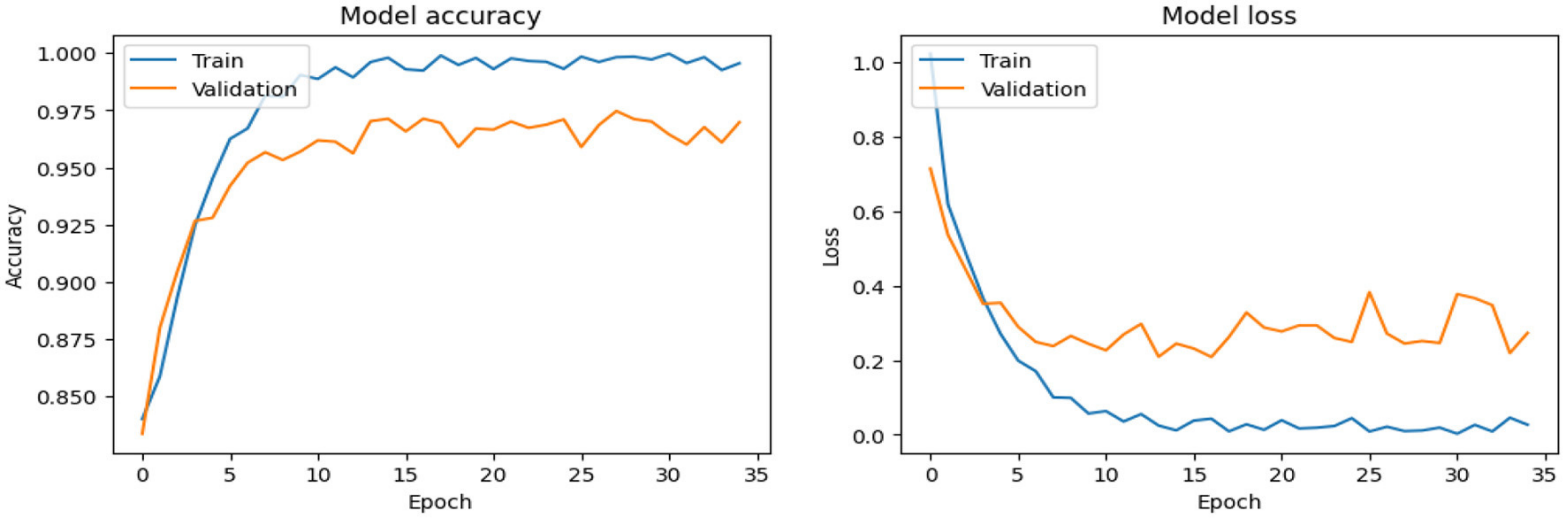
Analysis of the performance of the CNN model during training and validation.

**Figure 4:**
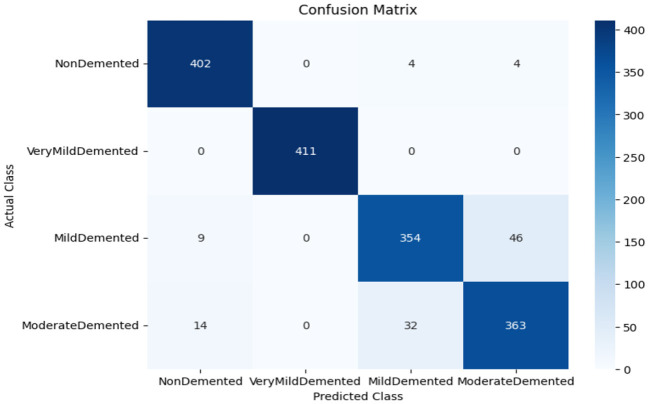
Confusion matrix of CNN model.

As shown in Supplementary Figure 1, the CNN model used for baseline feature extraction and classification consisted of an input layer of 128 × 128 × 3. It included four convolutional layers with filter sizes of 8, 16, 32, and 128, each followed by ReLU activation and max-pooling layers with a pool size of 2 × 2 and a stride of 2. The fully connected layers and ReLU activation were followed by an output layer with softmax activation for multi-class classification. The CNN model architecture achieved remarkable accuracy, reaching 93.35% accuracy on the validation dataset. The observed performance of accuracy and loss of the CNN model during training and validation is visualized in Figure 3. The confusion matrix is used to measure the CNN model performance across individual classes within the validation dataset as shown in Figure 4.

#### 4.5.2 Experiment 2: SECNN model

**Figure 5:**
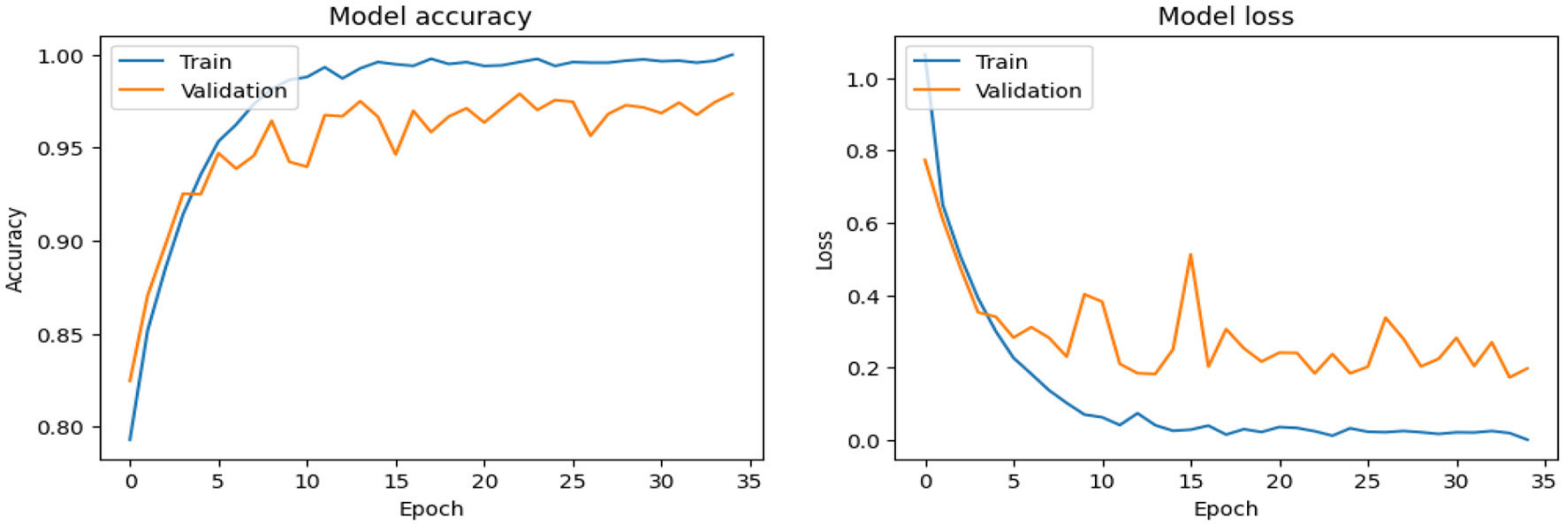
Analysis of the performance of the SECNN model during training and validation.

**Figure 6:**
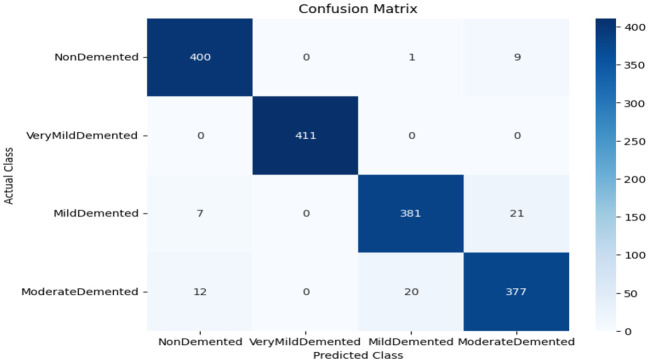
Confusion matrix of SECNN model.

As shown in Supplementary Figure 2, The SECNN model enhanced the CNN architecture by integrating Squeeze-and-Excitation (SE) blocks after each convolutional layer. Each SE block performed a squeeze operation using global average pooling to create a channel-wise feature vector, followed by an excitation operation with two fully connected layers using ReLU and sigmoid activations to generate reweighting coefficients. These coefficients were used to scale the feature maps. The architecture included four convolutional layers with SE blocks, using filter sizes of 8, 16, 32, and 128, followed by max-pooling layers, dense layers, and a softmax output layer. The model exhibited consistent improvement over the training epochs, converging to a final accuracy of 95.72% on the validation dataset. The observed performance of accuracy and loss of the SECNN model during training and validation is visualized in Figure 5. Also, Figure 6 displays the confusion matrix on the validation set of the SECNN model.

#### 4.5.3 Experiments 3: SECNN-RF

**Figure 7:**
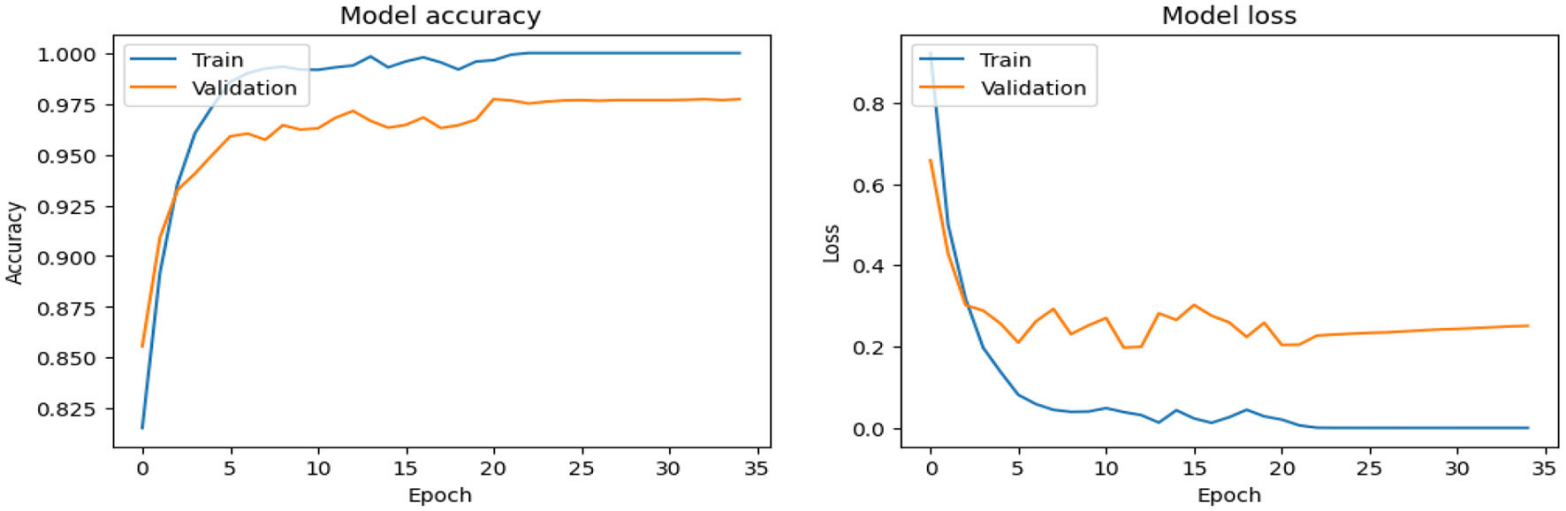
Analysis of performance of the proposed model during training and validation.

**Figure 8:**
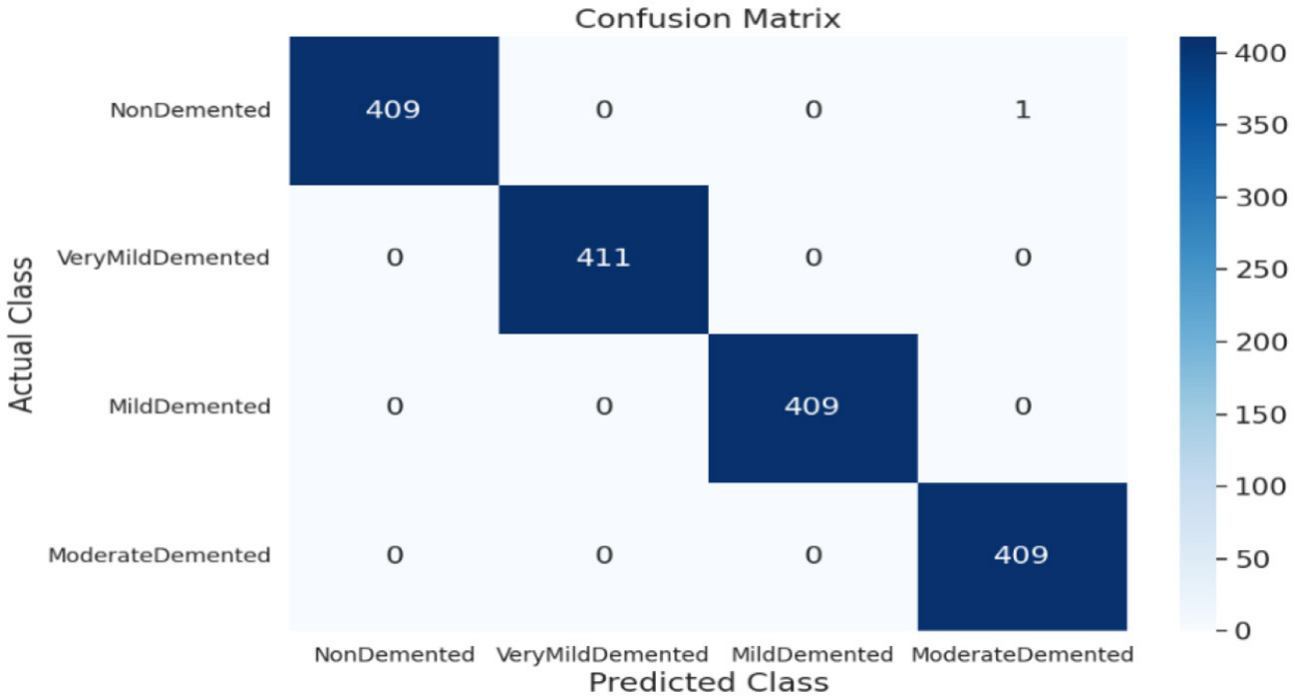
Confusion matrix of SECNN-RF proposed model.

As shown in Supplementary Figure 3, the SECNN-RF (proposed model) integrated the SECNN feature extractor with a Random Forest classifier. The SECNN, up to the final pooling layer, used four convolutional layers with filter sizes of 8, 16, 32, and 128, each followed by an SE block for enhanced feature extraction. Extracted features were then flattened and fed into the Random Forest classifier. The SECNN-RF model demonstrated exceptional accuracy, achieving 99.93% accuracy on the validation dataset. The observed performance of accuracy and loss of the proposed model during training and validation is visualized in Figure 7. The confusion matrix on the validation set of the proposed model (SECNN-RF) is presented in Figure 8.

#### 4.5.4 Experiment 4: varying SE-block ratios on SECNN-RF

**Figure 9:**
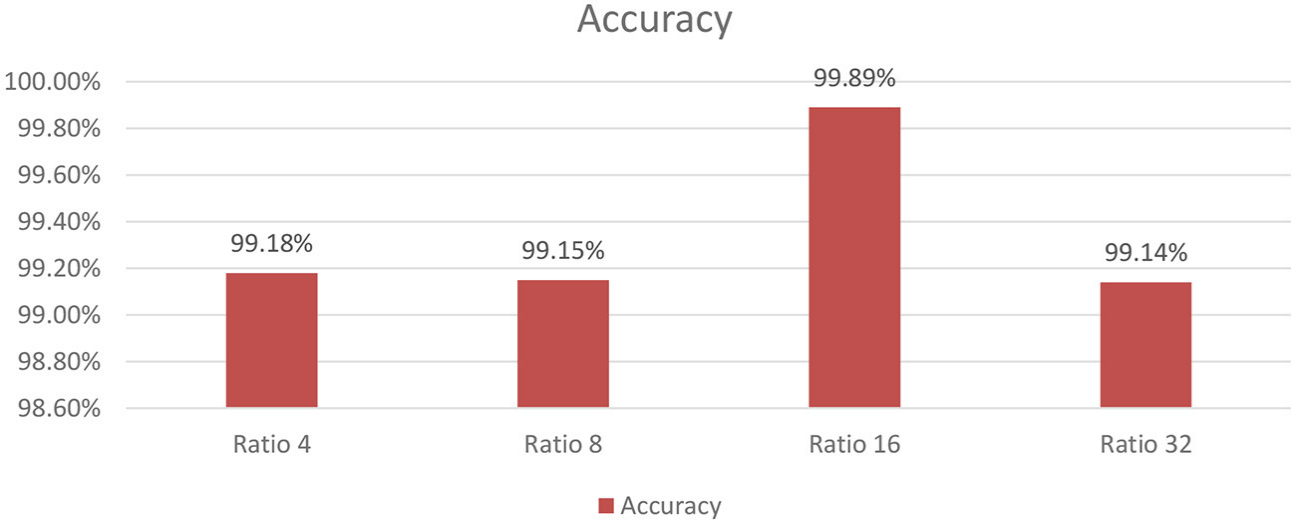
The analysis of varying SE block ratios.

As shown in Figure 9, the influence of varying the SE block ratio on the SECNN model was explored. Different SE block ratios (4, 8, 16, 32) were tested to observe their impact on model performance. The SE Block (Ratio = 4) with SECNN-RF model architecture achieved remarkable accuracy, reaching 99.18% accuracy on the test dataset. The SE Block (Ratio = 8) with SECNN-RF model architecture achieved remarkable accuracy, reaching 99.15% accuracy on the test dataset. The SE Block (Ratio = 16) with SECNN-RF model architecture achieved remarkable accuracy, reaching 99.89% accuracy on the test dataset. The SE Block (Ratio = 32) with SECNN-RF model architecture achieved remarkable accuracy, reaching 99.14% accuracy on the test dataset. The SE block with Ratio = 16 in the SECNN-RF model achieved the highest accuracy which equals 99.89 on the test set. The enhanced performance can be attributed to several factors, including the adaptive recalibration of channel-wise feature responses by SE blocks, which model the interdependencies between channels to emphasize informative features and suppress less useful ones, thereby enhancing feature representation. Additionally, the use of a reduction ratio of 1:16 strikes an optimal balance between model complexity and performance by effectively reducing the number of parameters added by the SE blocks. This balance allows the model to concentrate on critical features without overfitting, thus improving overall accuracy and robustness. Our SECNN-RF model contributes to minimizing trainable parameters. As shown in Table 4, there are variations in accuracy that depend on the variations in the number of trainable parameters, arising from the impact of different SE block ratios on the model's feature recalibration. SE blocks adjust the emphasis on feature maps, and different ratios affect how effectively important features are highlighted. The SE block ratio of 16 provided the optimal balance, achieving the highest accuracy of 99.89%, demonstrating that the choice of ratio significantly influences model performance.

**Table 4:** Trainable parameters and accuracy of SE block ratios.

**SE block ratio**	**Num. of trainable parameters**	**Accuracy**
4	744,058	99.18%
8	739,603	99.15%
**16**	**737,367**	**99.89%**
32	736,241	99.14%

The bold values indicate the best performance among the listed results.

#### 4.5.5 Experiment 5: varying ML algorithms on the SECNN model

**Figure 10:**
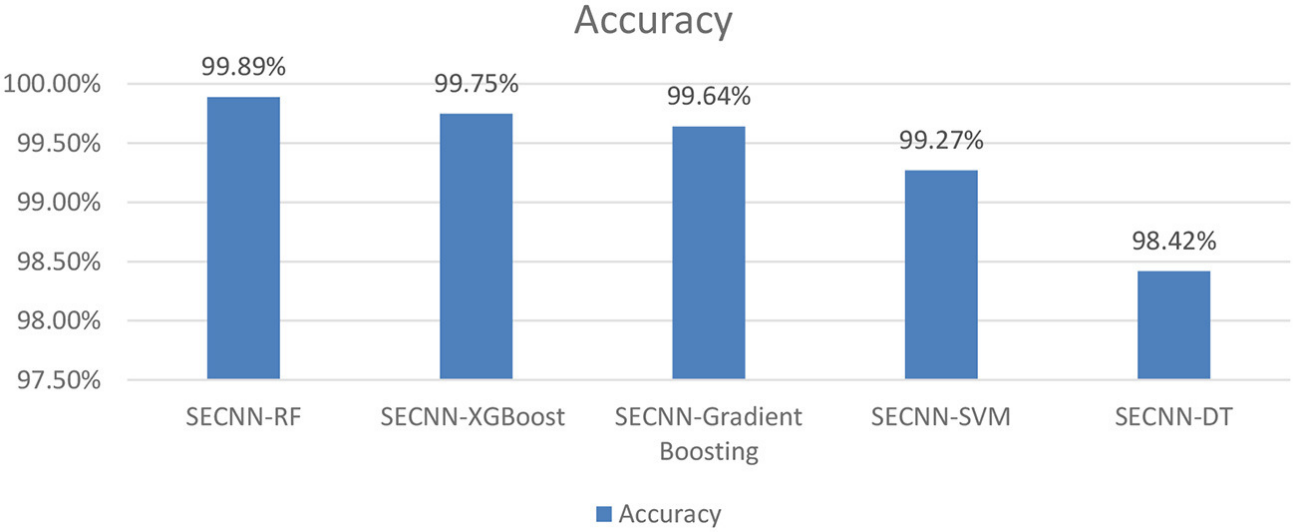
Analysis of varying ML algorithms on SECNN.

To further understand the impact of the choice of the classifier on the overall performance of the SECNN framework, we extended our ablation study by replacing the Random Forest classifier with other popular machine learning algorithms as shown in Figure 10. This evaluation aimed to compare the performance of different classifiers in leveraging the features extracted by the SECNN model for Alzheimer's Disease (AD) detection. We tested the following classifiers: Decision Tree (DT), Random Forest, Support Vector Machine (SVM), XGBoost, and Gradient Boosting. The accuracy of SECNN-RF, SECNN-XGBoost, SECNN-Gradient Boosting, SECNN-SVM, and SECNN-DT are 99.89, 99.75, 99.64, 99.27, and 98.42% respectively.

### 4.6 Ablation study

To accurately evaluate the efficacy of our proposed network architectures, this paper conducted a series of ablation experiments. This systematic approach enabled us to isolate the contributions of specific components and uncover their impact on task performance.

#### 4.6.1 Analysis of SECNN-RF (proposed model)

This subsection analyzes the performance of the SECNN-RF model. The SECNN-RF model demonstrated exceptional accuracy, achieving 99.93% accuracy on the validation set. This result indicates the effectiveness of the combined features in capturing discriminative information. Table 5 shows the proposed model (SECNN-RF) performance result based on the value of precision, recall, F1-score, and accuracy.

**Table 5:** The performance of the SECNN-RF model.

	**Precision**	**Recall**	**F1-score**	**Support**
Very mild demented	100.00%	100.00%	100.00%	411
Moderate demented	100.00%	100.00%	100.00%	409
Mild demented	99.90%	100.00%	99.89%	409
Non-demented	100.00%	100.00%	99.90%	410
Accuracy			99.93%	1,639
Macro avg	100.00%	100.00%	99.93%	1,639
Weight avg	99.90%	100.00%	99.93%	1,639

#### 4.6.2 Analysis of SECNN: remove random forest classifier

The Random Forest classifier was removed from the SECNN-RF framework. Instead of using the Random Forest for final classification, we reverted to the traditional approach of employing a dense layer with a SoftMax activation function to directly classify the features extracted by the CNN into the target categories. The CNN, equipped with SE blocks and Dropout layers, remains unchanged. The final fully connected layers are modified to culminate in a SoftMax layer, producing a probability distribution over the classes. The SECNN model architecture achieved remarkable accuracy, reaching 95.72% accuracy on the validation dataset. Table 6 displays the SECNN performance result based on the value of precision, recall, F1-score, and accuracy.

**Table 6:** The performance of the SECNN model.

	**Precision**	**Recall**	**F1-score**	**Support**
Very mild demented	100.00%	100.00%	100.00%	411
Moderate demented	92.24%	91.59%	91.28%	409
Mild demented	93.88%	92.51%	93.88%	409
Non-demented	96.66%	98.78%	97.71%	410
Accuracy			95.72%	1,639
Macro avg	95.70%	95.71%	95.70%	1,639
Weight avg	95.71%	95.72%	95.71%	1,639

#### 4.6.3 Analysis of CNN: remove SE-blocks and random forest classifier

Also, to investigate the contribution of the SE blocks to the model's performance, an ablation study was conducted by removing the SE blocks from the CNN architecture and removing the random forest classifier. The CNN model architecture achieved remarkable accuracy, reaching 93.35% accuracy on the validation dataset. Table 7 shows the CNN model performance result based on the value of precision, recall, F1-score, and accuracy.

**Table 7:** The performance of the CNN model.

	**Precision**	**Recall**	**F1-score**	**Support**
Very mild demented	100.00%	100.00%	100.00%	411
Moderate demented	87.89%	88.75%	88.32%	409
Mild demented	90.77%	86.55%	88.91%	409
Non-demented	94.59%	98.08%	96.29%	410
Accuracy			93.35%	1,639
Macro avg	93.31%	93.34%	93.30%	1,639
Weight avg	93.32%	93.35%	93.31%	1,639

Finally, the summary of the performance comparison is presented in Supplementary Table 2 which shows the evaluation metrics for three models: SECNN-RF, SECNN, and CNN on the validation dataset. Notably, the SECNN-RF model achieves the highest accuracy of 99.93%, showcasing its effectiveness in integrating Squeeze-and-Excitation (SE) blocks with a Random Forest classifier. This model benefits from adaptive feature recalibration and ensemble learning, demonstrating superior performance. The SECNN model follows with an accuracy of 95.72%, leveraging SE blocks to enhance feature representation, outperforming the baseline CNN model, which achieves an accuracy of 93.35%. These results underscore the significant enhancements in classification accuracy and robustness enabled by SE blocks and ensemble learning approaches.

### 4.7 Performance and confusion matrix analysis of experiment models on the test dataset

**Figure 11:**
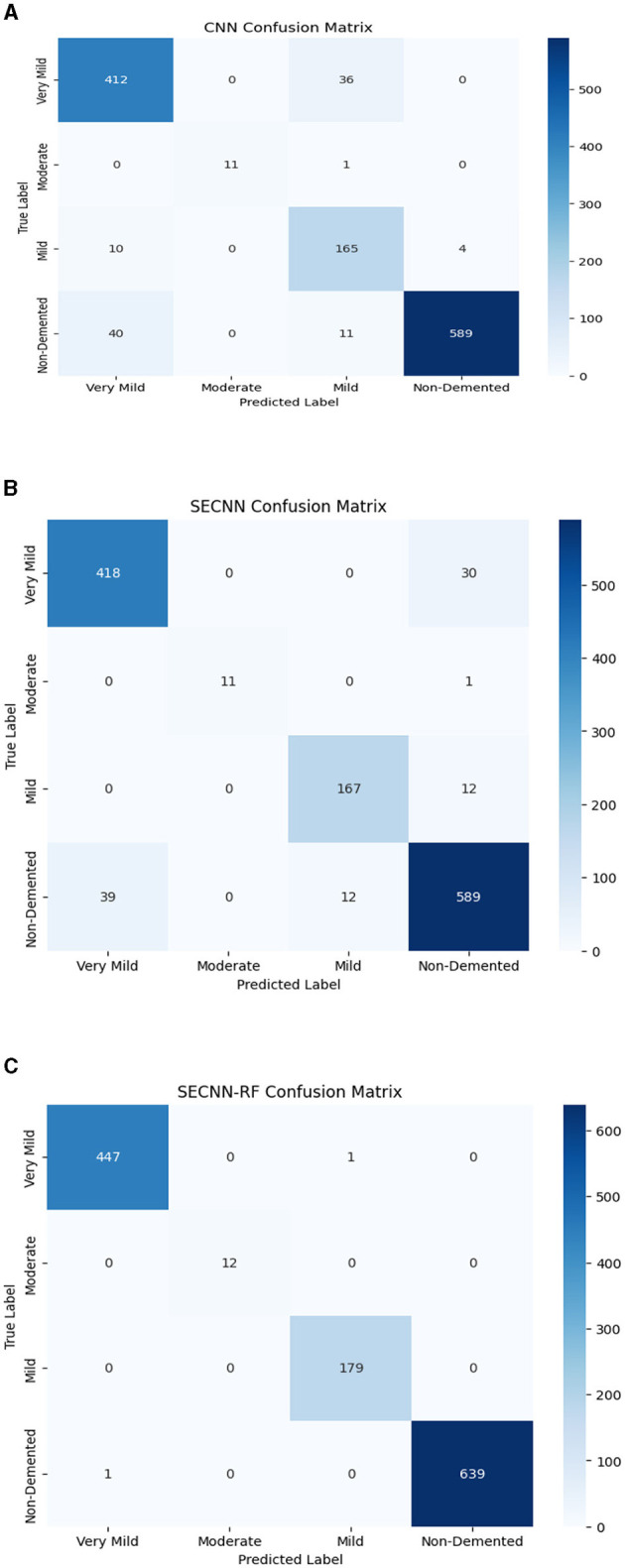
Confusion matrix of test dataset **(A)** CNN; **(B)** SECNN; **(C)** SECNN-RF models.

The confusion matrix serves as a fundamental tool for assessing and quantifying the performance of classification models. It provides a comprehensive breakdown of the model's predictions, offering insights into its effectiveness during the testing phase. we extensively analyzed the confusion matrix, represented in Figure 11, to measure the suggested model's performance across individual classes within the test dataset. Figure 11A shows the confusion matrix of the CNN model to detect demented cases on the test set. Figure 11B displays the confusion matrix on the test set of the SECNN model. Finally, Figure 11C shows the confusion matrix on the test set of the proposed model (SECNN-RF). Table 8 shows the evaluation metrics for experiment models. The SECNN-RF model achieves the highest accuracy of 99.89%, whereas the SECNN model follows with an accuracy of 92.65%, and finally, the baseline CNN model achieves an accuracy of 92.02%.

**Table 8:** The comparison of performance for experiment models on test dataset.

**Model**	**Class**	**Precision**	**Recall**	**F1-score**	**Support**	**Test accuracy**
**SECNN-RF (proposed model)**	Very mild demented	100.00%	100.00%	100.00%	448	**99.89%**
	Moderate demented	100.00%	100.00%	100.00%	12	
	Mild demented	99.80%	100.00%	99.80%	179	
	Non-demented	99.90%	99.80%	99.80%	640	
SECNN	Very mild demented	91.47%	93.30%	92.38%	448	92.65%
	Moderate demented	100.00%	91.67%	95.65%	12	
	Mild demented	93.30%	93.30%	93.30%	179	
	Non-demented	93.20%	92.03%	92.61%	640	
CNN	Very mild demented	89.18%	91.96%	90.55%	448	92.02%
	Moderate demented	100.00%	91.67%	95.65%	12	
	Mild demented	77.46%	92.18%	84.18%	179	
	Non-demented	99.33%	92.03%	95.54%	640	

The bold values indicate the best performance among the listed results.

### 4.8 Performance comparison with state-of-the-art models

Table 9 presents a performance comparison of our SECNN-RF against state-of-the-art models on the AD test dataset. All models were implemented under the same configurations with the same splitting dataset 80:20 between training and testing sets and also the same hyper-parameters such as dataset was trained using the Adam optimizer with a learning rate of 0.001, categorical cross-entropy loss, a batch size of 32, and for 35 epochs. Our proposed model achieved the highest accuracy by a significant margin. It is interesting to note that MOBILENET achieved the highest accuracy after our model of 95.29%, while RESNET50, a popular architecture in other domains, underperformed with an accuracy of 85.21% on the AD dataset. The proposed model achieved the highest performance compared with state-of-the-art models with an accuracy of 99.89% on the AD dataset. This implies that particular design decisions for the architecture could be essential to achieve the best performance on the classification tasks. These results show that SECNN-RF achieves the highest accuracy on the Alzheimer's MRI dataset while using fewer trainable parameters than or comparable to other models.

**Table 9:** Comparison between the proposed model with state-of-the-art models.

**Method**	**Tranable params**	**Accuracy (%)**	**Precision (%)**	**Recall (%)**	**AUC (%)**	**F1_Score (%)**
VGG16	15,763,908	92.71	86.14	84.45	97.73	85.27
VGG19	21,073,604	93.54	87.96	85.94	98.09	86.91
CNN	1,264,340	92.02	85.19	84.11	96.95	85.08
RESNET101	46,853,124	85.47	78.40	57.58	91.90	66.20
RESNET50	27,782,660	85.21	77.43	57.77	91.62	65.96
XCEPTION	25,056,428	91.89	84.68	82.50	96.94	83.57
MOBILENET	5,326,660	95.29	91.02	90.66	98.40	90.84
MOBILENETV2	4,880,068	92.99	86.03	85.90	96.34	85.96
INCEPTIONV3	23,036,644	90.50	84.32	76.02	96.41	79.85
DENSENET121	9,135,300	91.90	84.55	82.73	97.03	83.61
DENSENET169	16,051,396	93.50	87.31	86.52	97.78	86.92
Proposed Model (SECNN-RF)	737,367	99.89	99.70	99.85	99.89	99.75

### 4.9 Performance comparison with the existing research works

This section dives deep into a comparative analysis of our proposed model for AD detection, pitting it against both established models and a promising deep-learning approach from existing research as shown in Table 10. The authors in references (Muruganm et al., 2021; Ahmed et al., 2022; Bangyal et al., 2022; Loddo et al., 2022) divided the Kaggle dataset into training, validation, and testing sets with an 80:10:10 split. Conversely, the authors in references (Mohammed et al., 2021; Sharma et al., 2022; Balasundaram et al., 2023; El-Latif et al., 2023) utilized an 80:20 split between training and testing datasets.

**Table 10:** Comparison between the proposed model with other existing works models.

**References**	**Model**	**Tranable params**	**Accuracy (%)**	**Explainability**
Muruganm et al. (2021)	DEMentia NETwork	4,532,628	95.23	Yes
Loddo et al. (2022)	Ensemble Deep approach	–	97.71	No
Sharma et al. (2022)	Pretrained deep models	–	91.75	No
Mohammed et al. (2021)	Pretrained deep model + SVM	23,9 milions	94.80	No
Balasundaram et al. (2023)	Pretrained deep models	6,075 per image	94.10	No
Bangyal et al. (2022)	CNN	–	94.63	No
Ahmed et al. (2022)	DAD-Net	1,149,524	99.20	Yes
El-Latif et al. (2023)	Lightweight deep model	6,582,098	95.93	No
Our proposed model (Abdelaziz et al., 2024)	SECNN-RF	737,367	99.89	Yes

This paper leveraged the same dataset with the same splitting configuration between training, validation, and testing sets for a fair and insightful evaluation. A direct comparison across the various approaches presented in the previous table reveals the robust performance of our proposed model. While other methods have shown promise in their own right, our approach surpasses them in achieving the highest classification accuracy. Ahmed et al. (2022), while their work achieved a notable accuracy of 99.20, our model (SECNN-RF) surpasses their performance by 0.69% improvement in accuracy. Sharma et al. (2022), unfortunately, the approach yielded a significantly lower accuracy compared to both ours and other compared models that achieved an accuracy of 91.75%. These results highlight the effectiveness of our proposed model in tackling the classification task with superior accuracy. Our experiments demonstrate that SECNN-RF achieves high accuracy on the Alzheimer's MRI dataset while keeping the number of trainable parameters lower than or comparable to other models.

### 4.10 Explainability analysis

Explainability analysis is vital to comprehend how to accurately predict outputs and explain a conceptual approach. Currently, XAI is gaining popularity because of its interpretable mechanisms and ease of understanding. After classification performance accuracy and reliability had been assessed, explainability analyses were carried out by the saliency map explanation method. The Saliency Map is a well-known XAI technique used for evaluating prediction models. This study utilized the saliency map to explain the SECNN-RF model prediction outputs. This kind of map provides an accurate precise pixel-level interpretation of MRI scans, making them chiefly advantageous when high-resolution visions are essential for detailed image analysis. Apart from Grad-CAM, the saliency map generates coarse heatmaps according to the activation of a particular convolution layer, these maps make use of the gradient information to designate the most powerful pixels contributing to the model's prediction. This pixel-level granularity is critical for medical images, such as Alzheimer's Disease detection, where delicate patterns might be more revealing of the severity of the disease.

#### 4.10.1 Mild demented class

**Figure 12:**
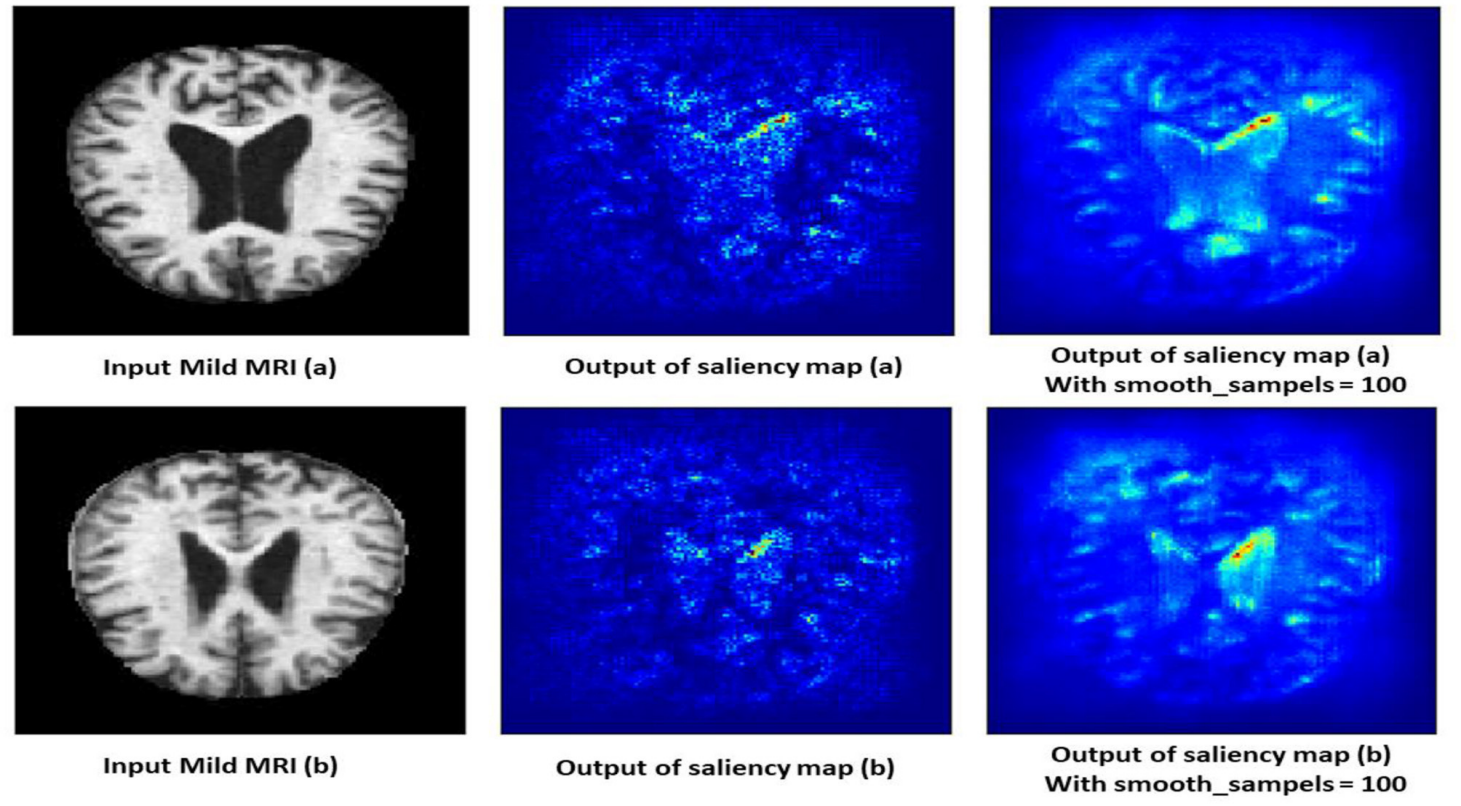
Saliency map estimated by SECNN-RF model for mild dementia class.

The saliency map for mild dementia highlights certain regions in the brain with varying intensities of color, indicating the areas that contributed most to the model's decision. As shown in Figure 12, the regions with higher intensity (red and yellow areas) are primarily located in the hippocampus and surrounding cortical areas. Mild dementia is often associated with atrophy in the hippocampus, a region crucial for memory and cognitive function. The model likely focuses on this area as it shows early signs of degeneration, and the highlighted cortical regions may indicate the model's attention to early cortical thinning, another characteristic of mild dementia.

#### 4.10.2 Moderate demented class

**Figure 13:**
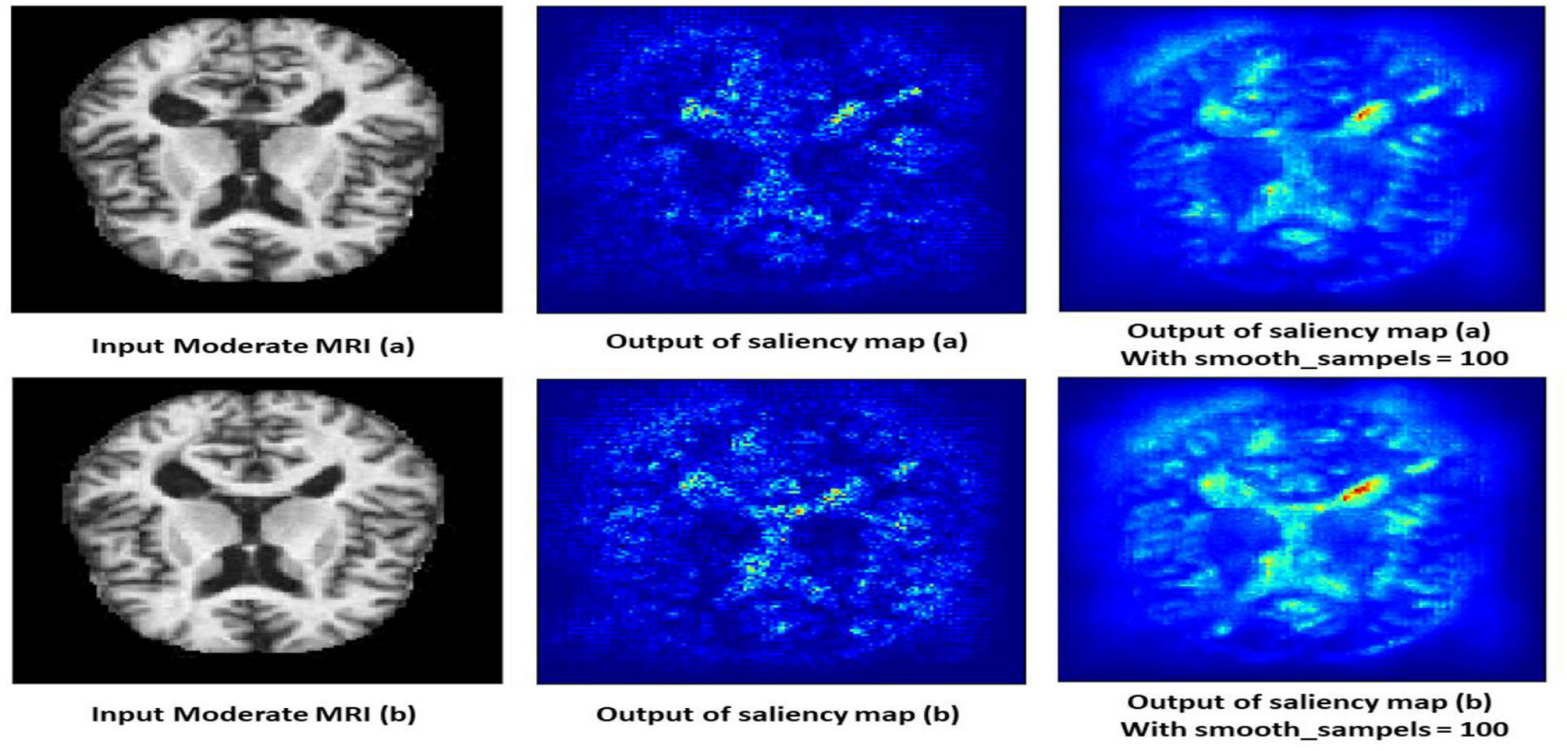
Saliency map estimated by SECNN-RF model for moderate dementia class.

The saliency map for moderate dementia shows more extensive and intense highlighted areas compared to mild dementia, particularly in the hippocampus and larger cortical regions as shown in Figure 13. The increased intensity and spread of the highlighted areas suggest more widespread brain changes associated with moderate dementia. Moderate dementia involves more severe atrophy of the hippocampus compared to mild dementia, which is reflected in the increased intensity in the saliency map. The model also focuses on larger areas of cortical degeneration, indicating that the disease has progressed beyond the initial stages and affects broader regions of the brain.

#### 4.10.3 Non-demented class

**Figure 14:**
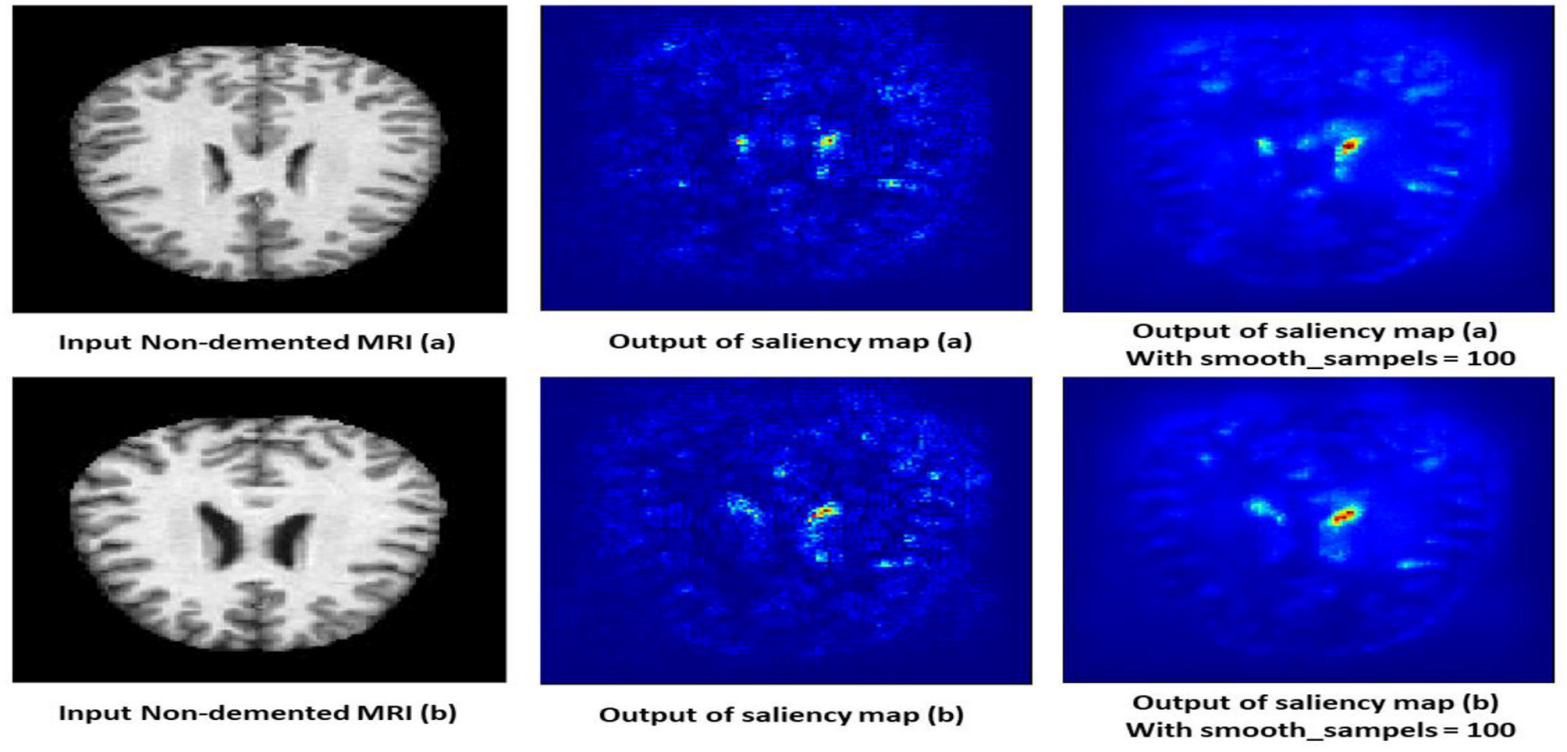
Saliency map estimated by SECNN-RF model for non-dementia class.

The saliency map for non-demented individuals shows minimal highlighted regions, indicating that the model found no significant abnormalities or changes associated with dementia. As shown in Figure 14, the intensity of the highlighted areas is low and uniformly distributed, reflecting a lack of focal points that the model associates with dementia. This absence of atrophy or cortical thinning aligns with the normal, healthy condition of a non-demented brain. The saliency map serves as a baseline, showing what a normal, non-demented brain looks like according to the model, which helps in contrasting with the saliency maps of demented cases.

#### 4.10.4 Very mild demented class

**Figure 15:**
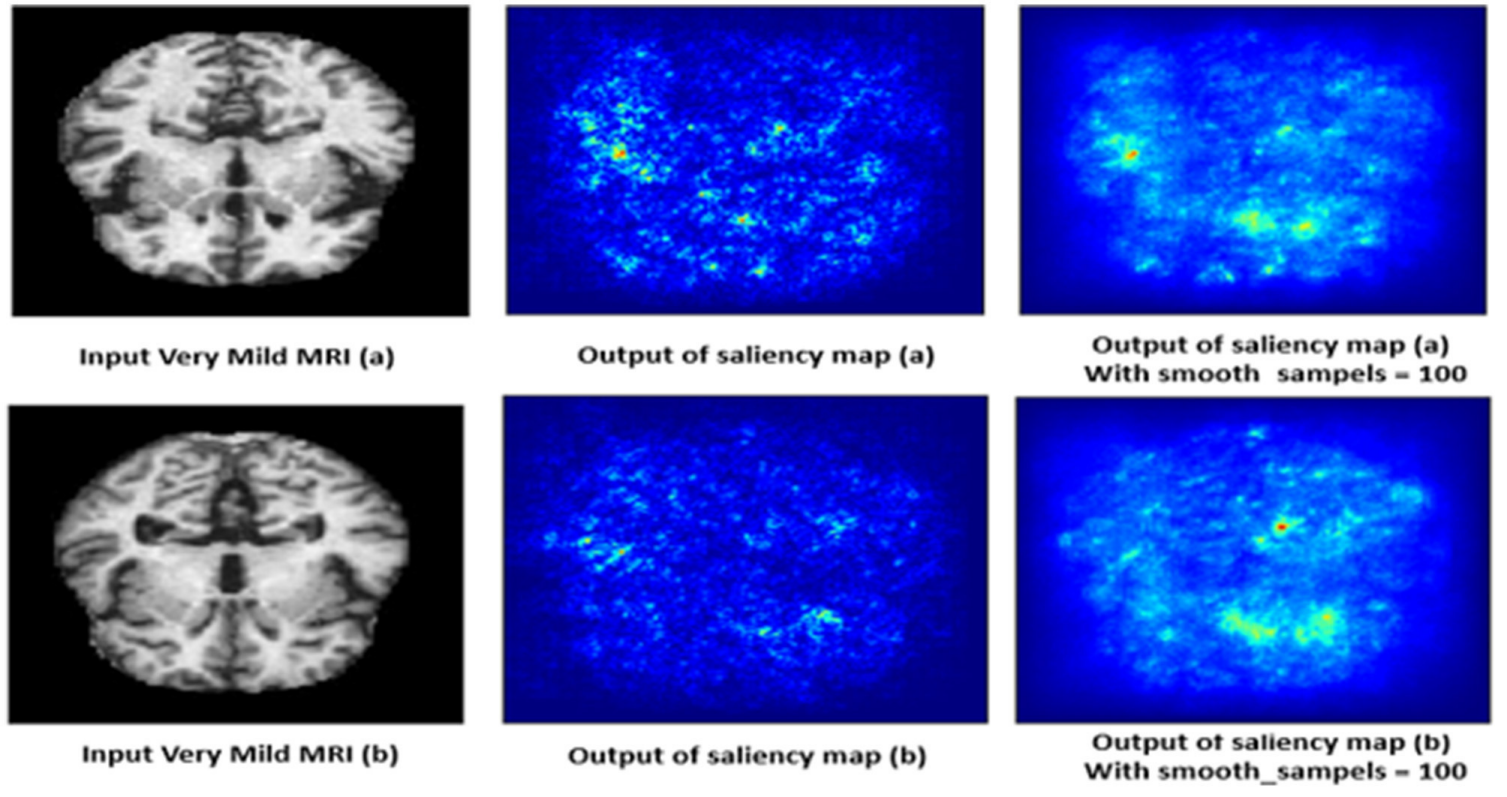
Saliency map estimated by SECNN-RF model for very mild dementia class.

The saliency map for very mild dementia shows subtle highlights, mainly in regions associated with early cognitive changes, such as the hippocampus and some cortical areas as shown in Figure 15. Compared to mild and moderate dementia, the highlighted regions are less intense, indicating early-stage changes that are less pronounced. These subtle changes are crucial for early diagnosis, as very mild dementia is characterized by initial signs of hippocampal atrophy and early cortical thinning. The model's ability to pick up on these subtle indicators helps in identifying the very early stages of dementia.

## 5 Strengths of the study

- The study's framework innovatively integrates SE blocks within each convolutional layer, applying an adaptive attention mechanism. This recalibrates channel-wise feature responses, allowing the network to dynamically prioritize the most informative features for Alzheimer's Disease (AD) detection from MRI scans. The use of SE blocks enhances the model's ability to capture critical patterns associated with AD.
- Unlike traditional CNN architectures that use a SoftMax layer for classification, the SECNN-RF framework employs a Random Forest classifier. This leverages the robust, ensemble learning capabilities of Random Forests, improving the model's classification accuracy and interpretability for AD diagnosis. The combination of SE blocks and Random Forests contributes significantly to the explainability of the model, providing insights into the importance of different features and straightforward interpretation of classification decisions.
- By utilizing a GlobalAveragePooling layer followed by feature flattening, the framework effectively condenses high-dimensional data into a manageable form. This ensures that the feature extraction phase is both computationally efficient and capable of capturing the essential characteristics of the input images.
- The SECNN-RF framework reduces trainable parameters without compromising performance, making it suitable for medical applications with limited computational resources. This balance between efficiency and accuracy is crucial for practical deployment in clinical settings.

## 6 Limitations of the study

- The dataset from Kaggle, while useful, may not be as comprehensive as others like the Alzheimer's Disease Neuroimaging Initiative (ADNI). Future studies could benefit from using more extensive and varied datasets.
- The study focuses on Alzheimer's Disease. Testing the framework on other medical conditions and imaging modalities would demonstrate broader applicability and robustness.
- The study only employs saliency maps to provide visual explanations of the model's decisions. Other advanced methods such as Grad-CAM, LIME, or SHAP were not utilized, which could provide more comprehensive and diverse insights into the model's decision-making process.

## 7 Conclusions

Our hybrid framework is termed a Squeeze-and-Excitation Convolutional Neural Network with Random Forest (SECNN-RF) represented for accurate image classification tasks and enhancing the interpretability of Alzheimer's disease (AD) images. SECNN-RF Achieves a remarkable 99.89% classification accuracy on the AD test dataset, surpassing state-of-the-art and existing work models. Leverages the SECNN-RF inherent attention mechanism to provide valuable insights into the model's decision-making process, fostering trust and transparency. The conducted ablation studies underscore the importance of each component, revealing that the integration of SE blocks and the choice of a robust classifier are pivotal in enhancing the model's performance. While achieving remarkable accuracy and interpretability, this work also opens doors for further research in explainable AI techniques for deeper disease understanding and personalized medicine. The authors encourage further exploration and development of such methodologies to pave the way for a future where AI empowers accurate diagnosis and individualized treatment plans, bringing hope to those impacted by neurodegenerative diseases.

## Data Availability

The original contributions presented in the study are included in the article/Supplementary material, further inquiries can be directed to the corresponding author.
