# Supplementary material for: Advanced interpretable diagnosis of Alzheimer's disease using SECNN-RF framework with explainable AI
Source: Front Artif Intell. 2024 Sep 2;7:1456069. doi: 10.3389/frai.2024.1456069 (PMC11402894; doi:10.3389/frai.2024.1456069)
Supplement: Supplementary file 1 [file Data_Sheet_1.docx]

**Supplementary Table** **1.** Model architecture and output shape for each layer.

| Layer (type) | Output Shape | Param # | Connected to |
| --- | --- | --- | --- |
| input_1 (InputLayer) | [(None, 128, 128, 3)] | 0 | [] |
| conv2d (Conv2D) | (None, 126, 126, 8) | 224 | ['input_1[0][0]'] |
| global_average_pooling2d (GlobalAveragePooling2D) | (None, 8) | 0 | ['conv2d[0][0]'] |
| reshape (Reshape) | (None, 1, 1, 8) | 0 | ['global_average_  pooling2d[0][0]'] |
| dense (Dense) | (None, 1, 1, 0) | 0 | ['reshape[0][0]'] |
| dense_1 (Dense) | (None, 1, 1, 8) | 0 | ['dense[0][0]'] |
| multiply (Multiply) | (None, 126, 126, 8) | 0 | ['conv2d[0][0]',  'dense_1[0][0]'] |
| dropout (Dropout) | (None, 63, 63, 8) | 0 | ['multiply[0][0]'] |
| max_pooling2d (MaxPooling2D) | (None, 61, 61, 16) | 0 | ['dropout[0][0]'] |
| conv2d_1 (Conv2D) | (None, 61, 61, 16) | 1168 | ['max_pooling2d[0][0]'] |
| global_average_pooling2d_1  (GlobalAveragePooling2D) | (None, 16) | 0 | ['conv2d_1[0][0]'] |
| reshape_1 (Reshape) | (None, 1, 1, 16) | 0 | ['global_average_  pooling2d_1[0][0]'] |
| dense_2 (Dense) | (None, 1, 1, 1) | 17 | ['reshape_1[0][0]'] |
| dense_3 (Dense) | (None, 1, 1, 16) | 32 | ['dense_2[0][0]'] |
| multiply_1 (Multiply) | (None, 61, 61, 16) | 0 | ['conv2d_1[0][0]',  'dense_3[0][0]'] |
| dropout_1 (Dropout) | (None, 61, 61, 16) | 0 | ['multiply_1[0][0]'] |
| max_pooling2d_1 (MaxPooling2D) | (None, 30, 30, 16) | 0 | ['dropout_1[0][0]'] |
| conv2d_2 (Conv2D) | (None, 28, 28, 32( | 4640 | ['max_pooling2d_1[0][0]'] |
| global_average_pooling2d_2  (GlobalAveragePooling2D) | (None, 32) | 0 | ['conv2d_2[0][0]'] |
| reshape_2 (Reshape) | (None, 1, 1, 32) | 0 | ['global_average_  pooling2d_2[0][0]'] |
| dense_4 (Dense) | (None, 1, 1, 2) | 66 | ['reshape_2[0][0]'] |
| dense_5 (Dense) | (None, 1, 1, 32) | 96 | ['dense_4[0][0]'] |
| multiply_2 (Multiply) | (None, 28, 28, 32) | 0 | ['conv2d_2[0][0]',  'dense_5[0][0]'] |
| dropout_2 (Dropout) | (None, 28, 28, 32) | 0 | ['multiply_2[0][0]'] |
| max_pooling2d_2 (MaxPooling2D) | (None, 14, 14, 32) | 0 | ['dropout_2[0][0]'] |
| conv2d_3 (Conv2D) | (None, 12, 12, 128) | 36992 | ['max_pooling2d_2[0][0]'] |
| global_average_pooling2d_3  (GlobalAveragePooling2D) | (None, 128) | 0 | ['conv2d_3[0][0]'] |
| reshape_3 (Reshape) | (None, 1, 1, 128) | 0 | ['global_average_  pooling2d_3[0][0]'] |
| dense_6 (Dense) | (None, 1, 1, 8) | 1032 | ['reshape_3[0][0]'] |
| dense_7 (Dense) | (None, 1, 1, 128) | 1152 | ['dense_6[0][0]'] |
| multiply_3 (Multiply) | (None, 12, 12, 128) | 0 | ['conv2d_3[0][0]'  'dense_7[0][0]'] |
| dropout_3 (Dropout) | (None, 12, 12, 128) | 0 | ['multiply_3[0][0]'] |
| max_pooling2d_3 | (None, 6, 6, 128) | 0 | ['dropout_3[0][0]'] |
| global_average_pooling2d_4  (GlobalAveragePooling2D) | (None, 128) | 0 | ['max_pooling2d_3[0][0]'] |
| flatten (Flatten) | (None, 128) | 0 | ['global_average_  pooling2d_4[0][0]'] |
| dense_8 (Dense) | (None, 1024) | 132096 | ['flatten[0][0]'] |
| dense_9 (Dense) | (None, 512) | 524800 | ['dense_8[0][0]'] |
| dense_10 (Dense) | (None, 64) | 32832 | ['dense_9[0][0]'] |
| dense_11 (Dense) | (None, 32) | 2080 | ['dense_10[0][0]'] |
| dense_12 (Dense) | (None, 4) | 132 | ['dense_11[0][0]'] |
| Trainable params: 737,367  No Non-trainable params: 0 | |  | |

**Supplementary Table 2.** The comparison of performance for experiment models on the validation dataset.

| Model | Class | Precision | Recall | F1-Score | support | Validation  Accuracy |
| --- | --- | --- | --- | --- | --- | --- |
| SECNN-RF  (Proposed model) | Very mild demented | 100.00 % | 100.00 % | 100.00 % | 411 | 99.93 % |
|  | Moderate demented | 100.00 % | 100.00 % | 100.00 % | 409 |  |
|  | Mild demented | 99.90 % | 100.00 % | 99.89 % | 409 |  |
|  | Non-demented | 100.00 % | 100.00 % | 99.90 % | 410 |  |
| SECNN | Very mild demented | 100.00 % | 100.00 % | 100.00 % | 411 | 95.72 % |
|  | Moderate demented | 92.24 % | 91.59 % | 91.28 % | 409 |  |
|  | Mild demented | 93.88 % | 92.51 % | 93.88 % | 409 |  |
|  | Non-demented | 96.66 % | 98.78 % | 97.71 % | 410 |  |
| CNN | Very mild demented | 100.00% | 100.00 % | 100.00 % | 411 | 93.35 % |
|  | Moderate demented | 87.89 % | 88.75 % | 88.32 % | 409 |  |
|  | Mild demented | 90.77 % | 86.55 % | 88.91 % | 409 |  |
|  | Non-demented | 94.59 % | 98.08 % | 96.29 % | 410 |  |


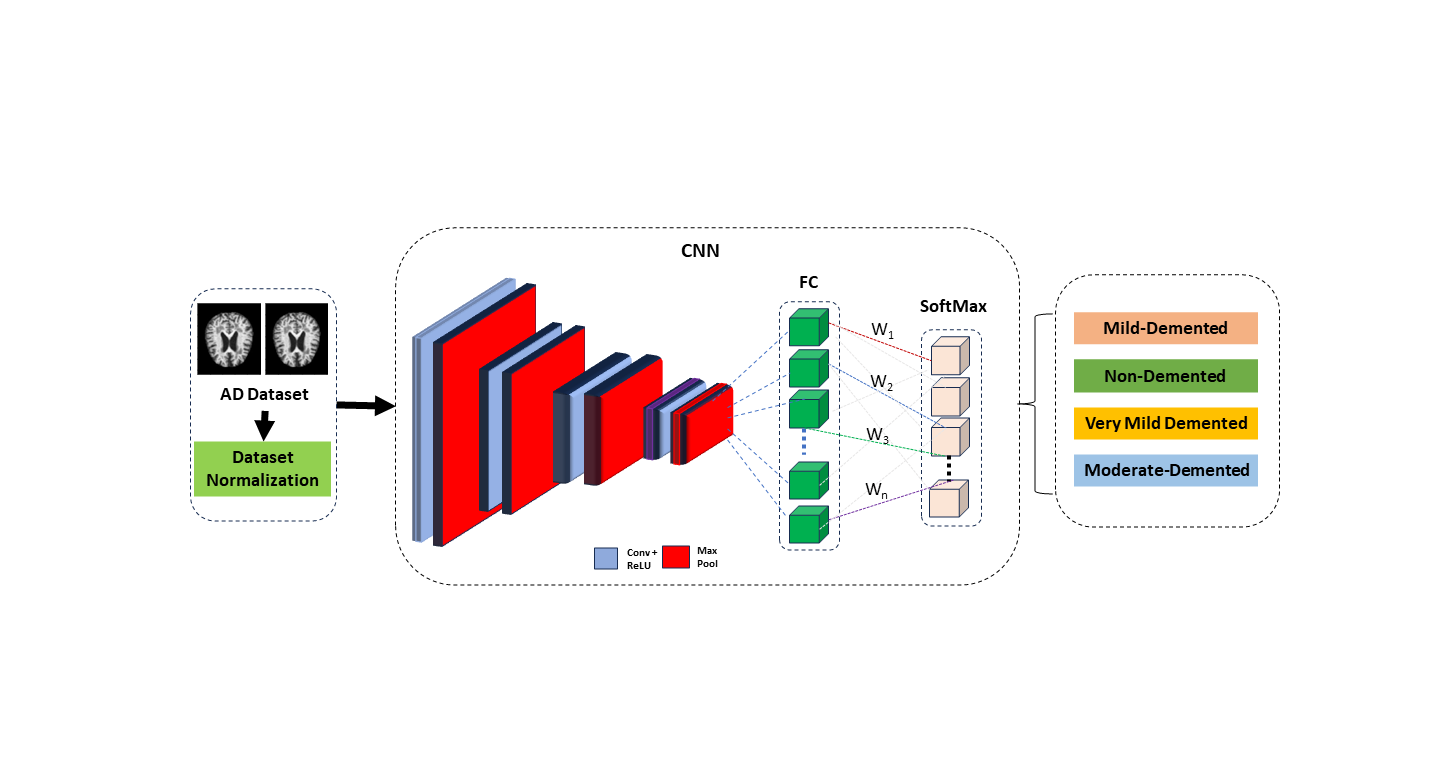


**Supplementary** **Figure 1.** The architecture of the CNN model.


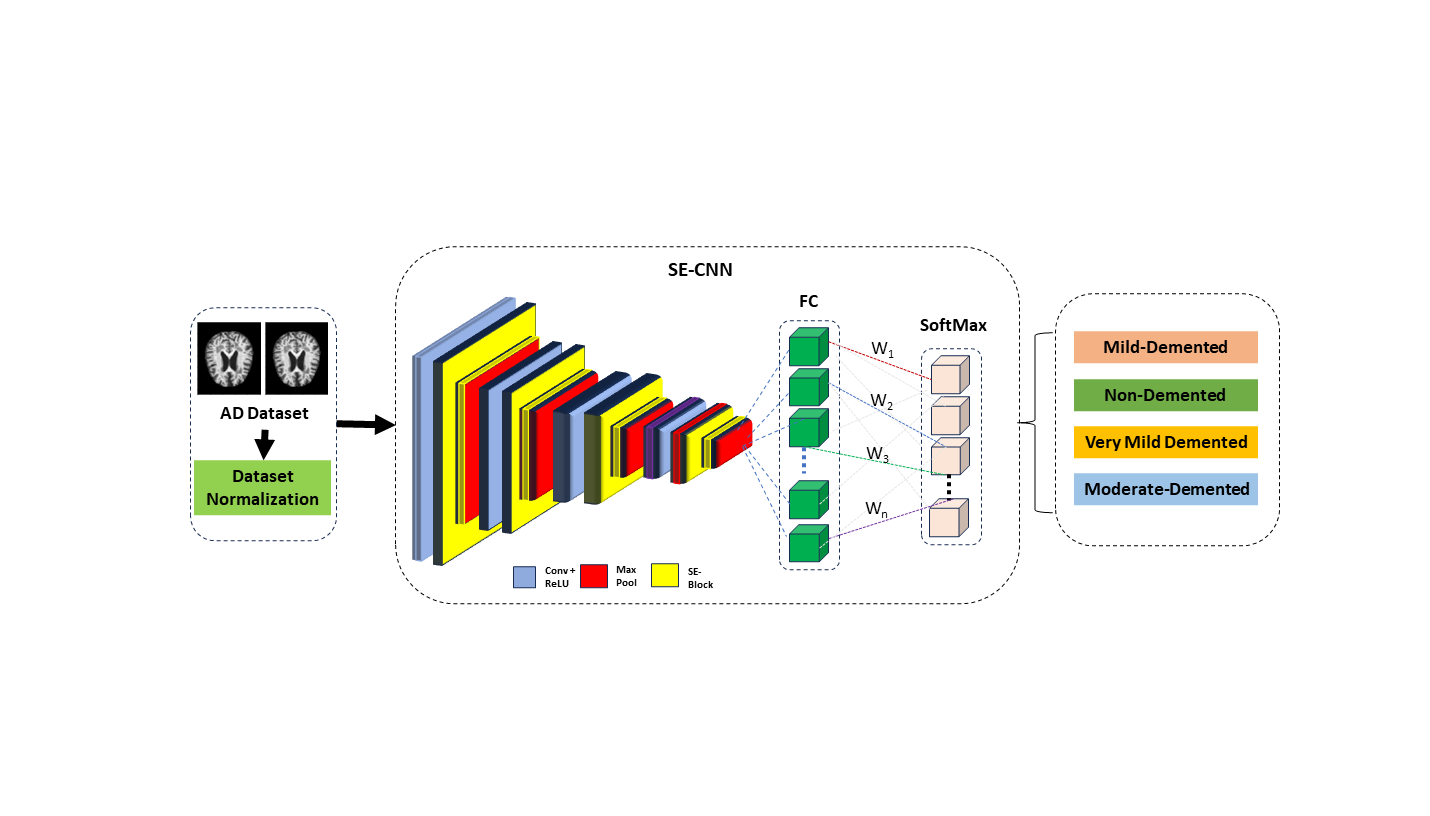


**Supplementary** **Figure 2**. The architecture of the SECNN model.


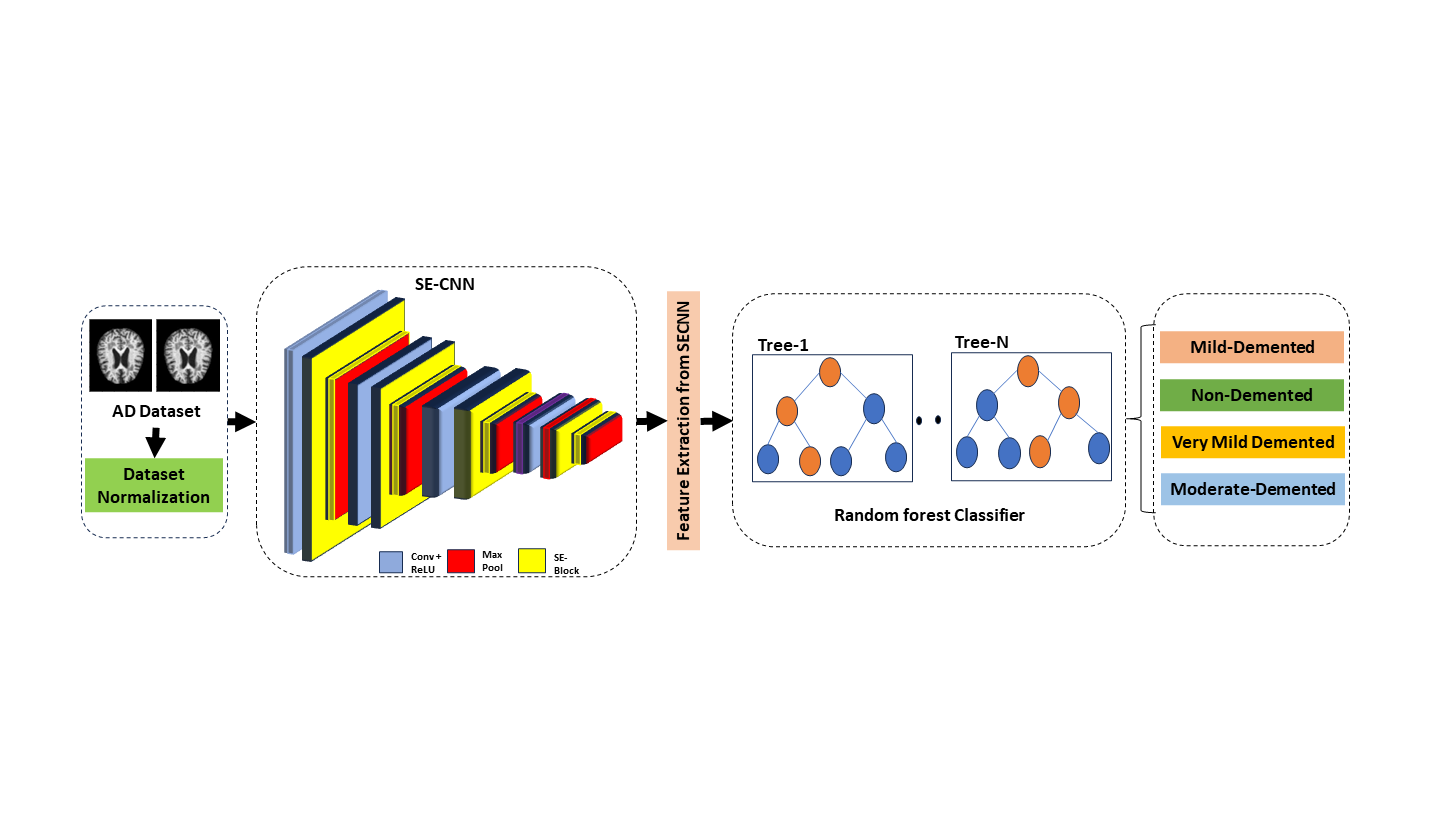


**Supplementary** **Figure 3.** The architecture of the SECNN-RF proposed model.
